# Numerical simulation and optimization of FTO/TiO_2_/CZTS/CuO/Au solar cell using SCAPS-1D

**DOI:** 10.1038/s41598-025-12999-0

**Published:** 2025-07-31

**Authors:** Lofty A. Lotfy, Mahmoud Abdelfatah, Swellam W. Sharshir, Ahmed A. El-Naggar, Walid Ismail, Abdelhamid El-Shaer

**Affiliations:** 1 Physics Department, Faculty of Science, Kafrelsheikh University, Kafrelsheikh, 33516 Egypt; 2 Nano Science and Technology Program, Faculty of Science, Kafrelsheikh University, Kafrelsheikh, 33516 Egypt; 3 Mechanical Engineering Department, Faculty of Engineering, Kafrelsheikh University, Kafrelsheikh, 33516 Egypt

**Keywords:** SCAPS-1D, Kesterite materials, Copper zinc tin sulfide (CZTS), Hole transport layer (HTL), Electron transport layer (ETL), Materials for optics, Renewable energy

## Abstract

Kesterite materials, especially copper zinc tin sulphide (CZTS), have emerged as very promising solar cell materials because of their sustainability, cost-effectiveness, and environmentally friendly composition. CZTS, composed of abundant and nontoxic elements, stands as a leading candidate among materials for efficient, sustainable, and cost-effective photovoltaic technologies. The " FTO/TiO_2_/CZTS/CuO/Au " solar cell has been simulated using SCAPS-1D, where FTO is the front contact, TiO_2_ is the electron transport layer, CZTS is the absorber layer, CuO is the hole transport layer and Au is the back contact, this device presenting an investigation of the structure, material properties, and carrier dynamics of such a device under standard AM 1.5 G illumination at 300 K. By defining characteristics of the layers, such as thickness, band gap, doping concentrations, and mobility, the software gives insight into photovoltaic performance with main results concerning J-V curves, quantum efficiency, and energy band diagrams. The maximum simulated efficiency achieved is 33.56% by optimising different parameters such as thickness, carrier concentration, and band gap.

## Introduction

Solar energy constitutes one of the most important renewable resources that, in principle, could provide a solution to the global energy crisis^1^. In the last decade, the global photovoltaic device market has rapidly grown, with the capacity of installed devices increasing from 6.1 GW in 2006 to 291 GW in 2016^2^. While the active semiconductor material layers are thinner than crystalline wafers, the capability of depositing films on thin substrates enables flexible PV modules to be manufactured with potentially lower material and manufacturing costs using high-throughput deposition processes^3^.

There are two classes of semiconductors: elemental semiconductors which include silicon or germanium and are widely used in the electronics industry and compound semiconductors which include metal oxides, chalcogenides, some of which have photocatalytic properties^4^.

Metal oxide semiconductors (MOs) are good candidates for the use in photovoltaic (PV) cells as they are known to be inexpensive, non-toxic, chemically stable, and suitability for ambient deposition^5,6^. These materials are also used in a variety of commercial applications, both as active materials, for example, in transistors of active matrix displays^7^, and passive ones, like transparent conductive electrodes and charge transport layers in solar cells^8^. Recently, these materials have attracted a lot of attention as they form all-oxide PV cells, based on heterojunctions where MOs are the only active component in the device, and they are believed to reduce the cost of producing PV, due to their low cost and ease of manufacture^8^. All-oxide PV cells, which are entirely based on MOs and rely on heterojunctions, have recently gained significant attention due to their potential to lower PV costs, thanks to their affordability and simple production processes^9,10^. Also, kesterite specific quaternary semiconductors containing elements for instance, CIGS, CdTe, and CZTS have also been strongly considered as ideal for tackling this problem. This is because these materials have more than good enough photovoltaic characteristics^11,12^.

Photoelectric solar cell systems are customarily designed with a heterojunction construct where FTO is employed as the front contact, Au as the back contact, CuO as the n-type layer, CZTS for the light absorbing layer and TiO₂ for the p-type layer. The structure is prevalent in solar cells and photocatalytic materials that make use of these materials to efficiently convert sunlight into electrical energy^13,14^.

Due to its semi-transparency in the visible range and high refractive index (2.2–2.4), titanium dioxide has great promise for use in both electronics and optoelectronics projects^15^. TiO₂ is widely recognized for its remarkable characteristics and a band gap of over 3 eV. Great attention has been paid to TiO₂-based dye-sensitized solar cells due to their high efficiency^16^, as the efficiency has been reported to exceed 11% in dye-sensitized solar cells^17^.

Copper zinc tin sulfide is a quaternary semiconductor that goes by the abbreviation CZTS and is of particular interest owing to its attractive optoelectronic properties and the multiplicity and environmental friendliness of its constituent elements^18^. CZTS has a direct band gap of approximately 1.5 eV and has an excellent absorption coefficient of 10^4^ cm^− 1^, thus rendering the material suitable for its application as a thin film photovoltaic cell^19^. Various deposition techniques that are used to prepare CZTS include sputtering, vacuum co-deposition, electro-deposition, spin-coating, dip-coating, pulsed laser deposition, and chemical bath deposition^20^. These materials are low-cost and effective and are suitable for optimizing the thin films CZTS as the light-absorbing layer in solar cells^21^. Significant material limitations in CZTS solar cells have been identified, such as deep-level traps, which negatively affect the efficiency of the cells^22^. These traps have been addressed through heat treatment in an oxygen-rich environment, which has proven effective in improving the efficiency of Cu_2_ZnSnS_4_ solar cells^23^. Regarding performance bottlenecks, recent reviews have focused on strategies to enhance efficiency in CZTSSe solar cells, with attention given to limitations such as low V_oc_, J_sc_, and FF^22^. Additionally, the use of cadmium-free buffer layers in kesterite thin-film solar cells has been explored, highlighting their potential to improve performance while reducing environmental impact^24^.

Cupric oxide (CuO) is an excellent candidate because of its peculiar properties that include stability and high absorption coefficient within the visible light range. It is generally a p-type direct bandgap semiconductor with a bandgap energy of about 1.4 eV^25^. CuO nanostructures can be synthesized using different methods and techniques to obtain specific morphologies and shapes, which makes them suitable for a wide range of applications of its kind, including antimicrobial, photocatalysis, batteries, solar cells, light-emitting diodes, and gas sensors^26^.

The current exercise involves^27,28^ the usage of SCAPS-1D solar cell simulator capacitance program for the analysis of a highly efficient n-i-p Charge-Transport Materials (CPs) model solar cell. SCAPS software is specifically made for simulating one-dimensional solar cell operation, specifically heterojunction and especially thin-film photovoltaic cells, which enable very accurate modelling and simulation^29^. This work presents SCAPS numerical simulations for FTO/CuO/CZTS/ TiO₂/Au heterojunction solar cell systems. The focus will be on investigating how the bandgap, carrier concentration, and thickness of the window and absorber layers influence the key characteristics of the solar cell. The findings from these simulations will serve as an important initial step in identifying the optimal conditions for producing high-efficiency solar devices.

## Structure of the apparatus and simulation approach

**Fig. 1:**
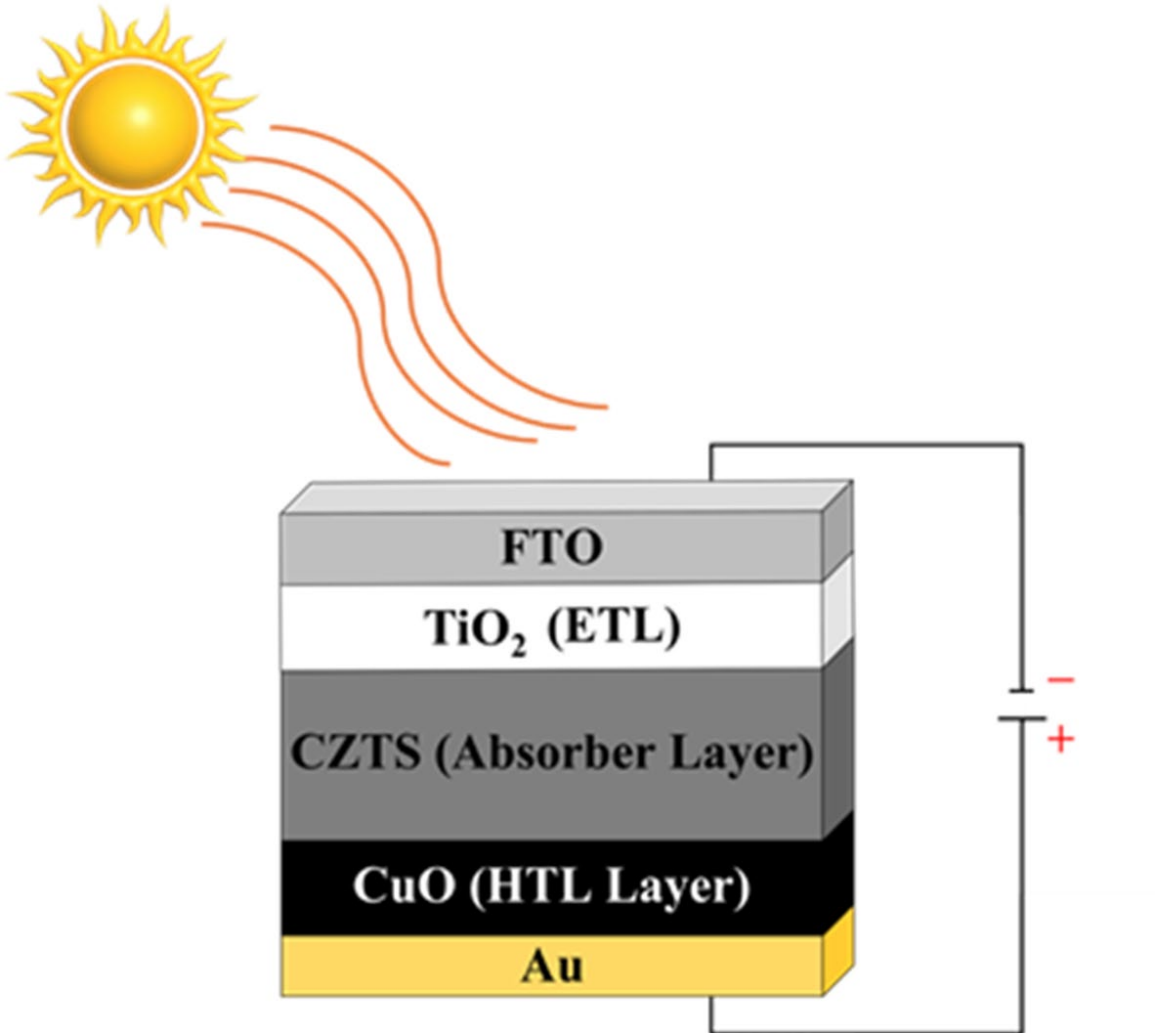
Schematic diagram of the FTO/TiO_2_/CZTS/CuO/Au heterojunction solar cell.

The Charge-Transport Materials (CPs)-based n-i-p planar kesterite device models were software (Solar Cell Capacitance Simulator, version 3.3.07) for this study. Inorganic n-i-p CPs heterojunction solar cells consist of an electron transport layer (‘n’-TiO_2_ thin film) and an active absorbing layer (CZTS) with a hole transport layer, p-CuO thin film. These all would be modeled to solve Poisson and continuity equations. The transparent conductive oxide (FTO) serves as front contact, while gold (Au) acts as the back contact. A schematic diagram of the heterojunction device structure used in the simulation is shown in Fig. 1. SCAPS-1D allows for the simulation of multi-layer systems, incorporating parameters such as material properties, doping concentrations, energy band structures, and defect densities. This allows for the exploration of how defects, especially those present in the ETL, alter charge carrier recombination, transport, and the efficiency of a solar cell. The output of this computation included band diagrams, quantum efficiency (QE), current-voltage (I-V) characteristics and other salvaged facts regarding performance of the device such as open-circuit voltage (V_oc_), short-circuit current density (J_sc_), fill factor (FF), and efficiency (η). These findings are important for improvements of design and efficiency of solar cells^30,31^. The optimized parameters and the structure of the kesterite solar cell applied in this work are mentioned in the Tables 1 and 2; Fig. 1, respectively.

**Table 1:** Parameters for the different layers of proposed solar cell^32–35^.

Parameter of the material	CuO	CZTS	TiO_2_
Thickness (nm)	50	1500	50
Dielectric permittivity	18.1	9.0	10
Bandgap (eV)	1.2	1.4	3.2
Electron affinity (eV)	4.07	4.1	4.2
Effective density of states of valence band maximum (cm^−3^)	5.5 × 10^20^	1.8 × 10^18^	6 × 10^7^
Effective density of states of conduction band minimum (cm^−3^)	2.2 × 10^19^	2.2 × 10^18^	2 × 10^7^
Acceptor concentration (cm^−3^)	1.0 × 10^18^	1.0 × 10^19^	0
Donor concentration (cm^−3^)	0	0	1 × 10^17^
Mobility of hole (cm^2^/(V s))	20	1.0 × 10^2^	2.5 × 10^2^
Mobility of Electron (cm^2^/(V s))	200	1.25 × 10^1^	1 × 10^2^
Electron thermal velocity (cm/s)	10^7^	10^7^	10^7^
Hole thermal velocity (cm/s)	4.6 × 10^6^	10^7^	10^7^
Deffect density N_t_ (cm^−3^)	1 × 10^15^	1 × 10^15^	1 × 10^16^

**Table 2:** The contact parameters in the simulation^36,37^.

Contacts	Front metal contact properties (FTO)	Back metal contact properties (Au)
Metal work function (eV)	4.07	4.98
Surface recombination velocity of electron (cm/s)	1.000 × 10^7^	1.000 × 10^7^
Surface recombination velocity of hole (cm/s)	1.000 × 10^7^	1.000 × 10^7^

## Result and discussion

### Impact of cupric oxide as hole transport layer on solar cell performance

By acting as the hole transport layer (HTL), cupric oxide (CuO) effectively enhances the efficiency (η) of kesterite solar cell by acting as an effective pathway for hole transport and allowing hole injection from the kesterite layer to the electrode^38^. Cupric oxide’s high hole mobility and suitable energy alignment with kesterite materials, such as (CZTS, CZTSSe, Cu_2_ZnSn (S_x_Se_1−x_)) materials, make this efficient. This alignment improves the photocurrent and reduces recombination losses, which improve the solar cell’s total efficiency^39^.

#### Influence of thickness of cupric oxide (CuO) on the open-circuit voltage (V_oc_), short circuit current density (J_sc_), fill factor (FF%), and efficiency (η)

The parameters such as thickness, band gap and the acceptor concentration of cupric oxide (CuO) have a significant impact on the solar cells performance^40^. An optimal CuO thickness improves open-circuit voltage (V_oc_) and reduces recombination, while a too thick CuO layer can introduce resistive losses and lower V_oc_ and short-circuit current density (J_sc_).

**Fig. 2:**
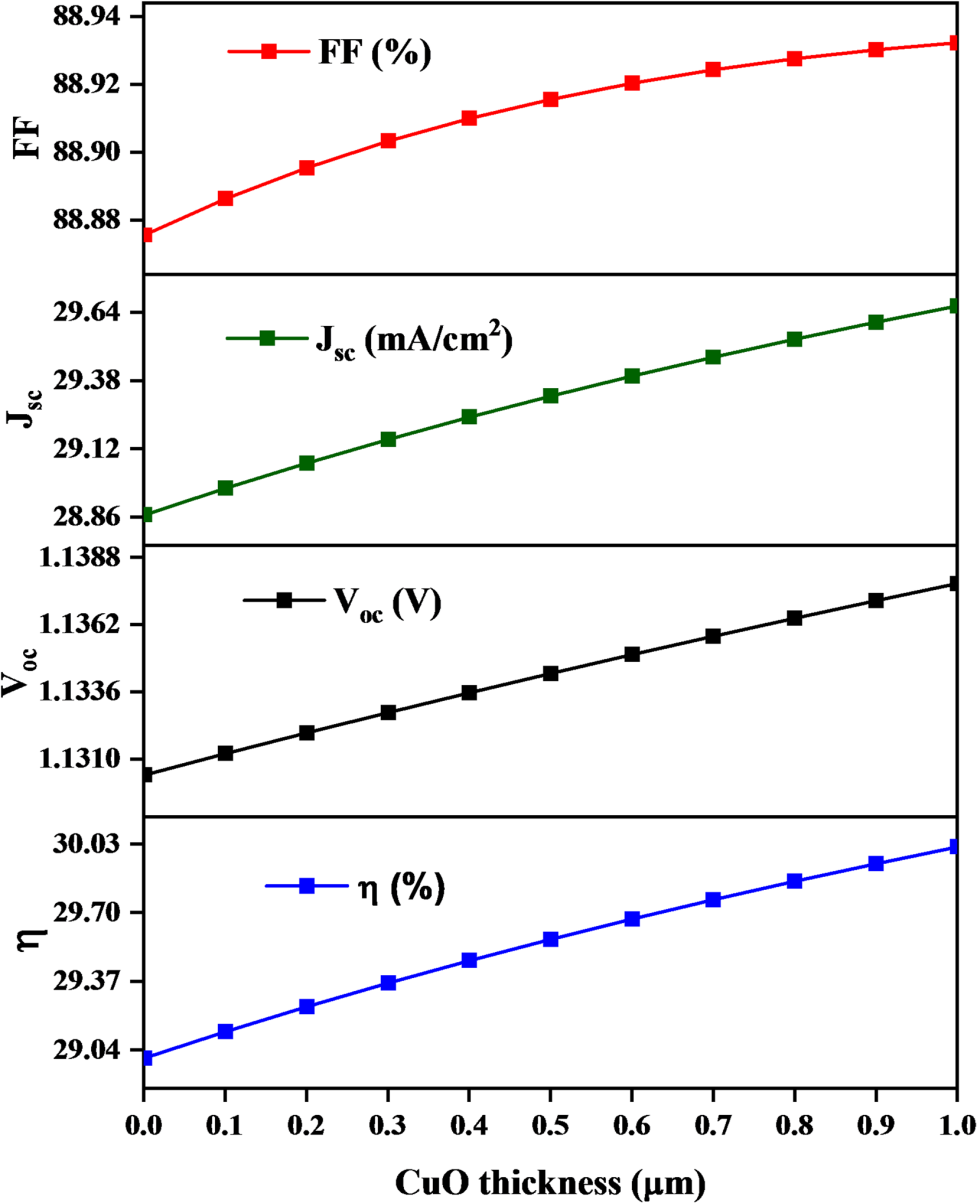
Shows the relationship between CuO thin film thickness and the solar cell parameters.

The increase in PCE from 29.04 to 30.03%, V_oc_ from 1.1306 to 1.1378 V, J_sc_ From 28.86 to 29.64 mA/cm^2^ and FF increase from 88.87 to 88.93 are found by increasing the CuO HTL thickness from 0.1 to 1 μm. It can be observed that the PCE, V_oc_, J_sc_, and FF increase by increasing the thickness due to by increasing CuO HTL thickness improves hole transport, reduces recombination, and boosts efficiency and stability. The results confirm that the ideal thickness to achieve the highest efficiency in this device is 1 μm (Figure 2).

#### Impact of cupric oxide (CuO) thickness on band gap (E_g_) variation

This section examines how changes in the thickness and band gap of the p-CuO (HTL) affect the device’s performance. The band gap of p-CuO HTL varied between 1.1 and 1.7 eV, and the thickness ranged from 0 to 1 μm. Throughout this study, the acceptor concentration in the CuO HTL was held constant at 10^18^ cm⁻³, as detailed in Table 1.

**Fig. 3:**
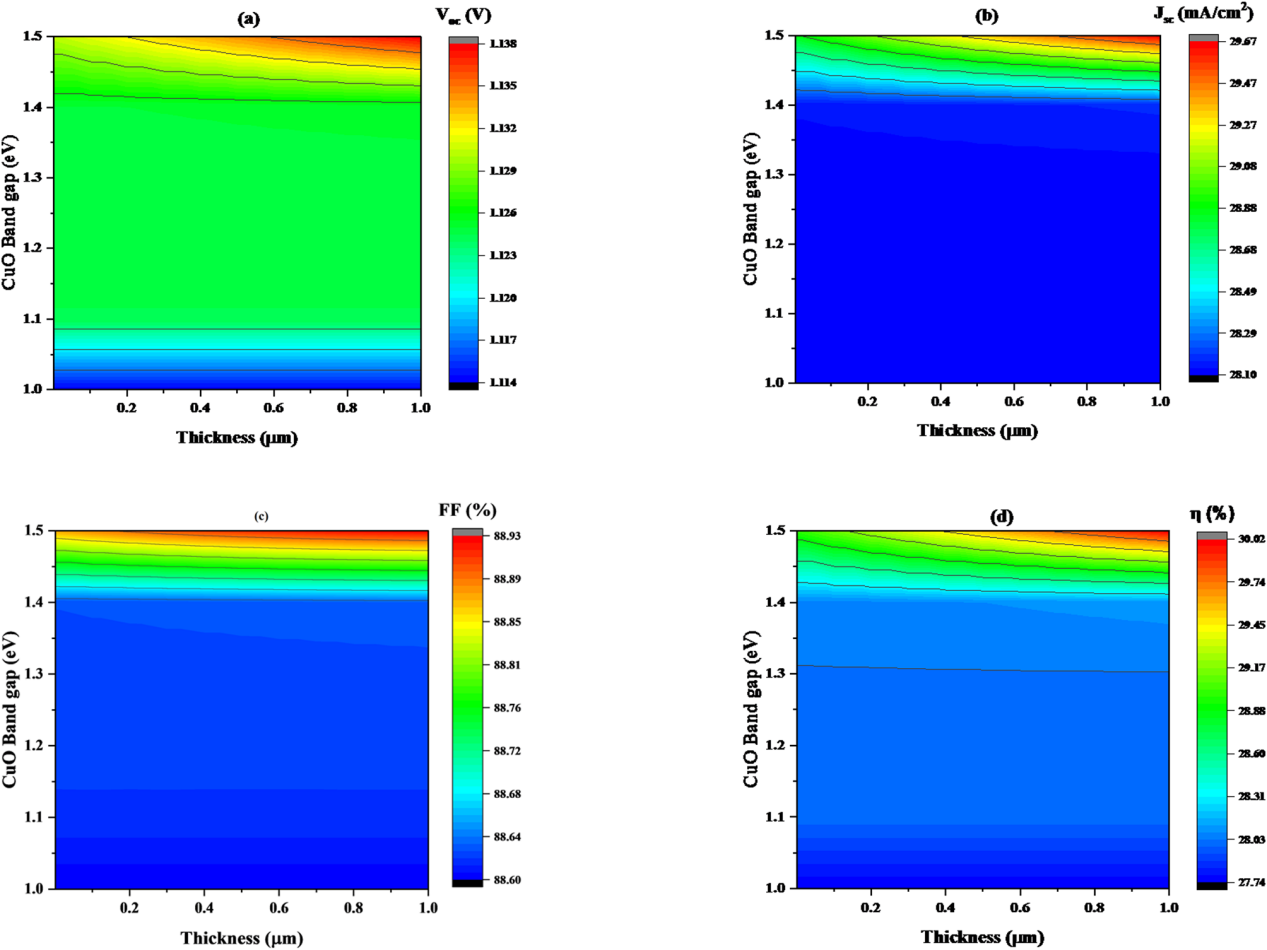
Illustrates the change in solar cell performance with variation in band gap/thickness of CuO HTL for a given value of acceptor concentration 10^16^ cm^−3^, where (**a**) V_oc_, (**b**) J_sc_, (**c**) FF, and (**d**) η.

According to Fig. 3**(a)**, it can be observed that the open-circuit voltage (V_oc_) values tend to be lowest at smaller band gap values as the thickness increase. However, at a band gap of (1.45 eV), V_oc_ begins to increase, reaching its maximum value of (1.139 V) at a thickness of (1 μm) and a band gap of (1.5 eV).

In Fig. 3**(b)**, J_sc_ values are also lowest at smaller band gap values as the thickness increases. At a band gap of (1.474 eV), J_sc_ begins to increase, ultimately reaching its maximum value of 29.71 mA/cm² at a thickness of 1 μm and a band gap of 1.5 eV.

As shown in Fig. 3**(c)**, FF values tend to be lowest at smaller band gap values as the thickness increases. However, at a band gap of 1.4704 eV, FF begins to increase, ultimately reaching its maximum value of 88.94% at a thickness of 1 μm and a band gap of 1.5 eV.

According to Fig. 3**(d)**, it can be observed that the efficiency (η)values tend to be lowest at smaller band gap values as the thickness increase. However, at a band gap of (1.467 eV), η begins to increase, ultimately reaching its maximum value of (30.03%) at a thickness of (1 μm) and a band gap of (1.5 eV). The results show that the ideal thickness and band gap to achieve the highest efficiency in this device is 1 μm and 1.5 eV, respectively.

#### Impact of cupric oxide (CuO) thickness on carrier (acceptor) concentration variation

In this section, the influence of thickness and concentration variations of the p-CuO HTL on the performance of the device has also been studied. The carrier concentration of the p-CuO HTL was adjusted within the range of 10^14^ to 10^21^ cm^− 3^, while the thickness was varied from 0 to 1 μm. The band gap energy of CuO HTL was still constant at 1.2 eV, as shown in Table 1.

**Fig. 4:**
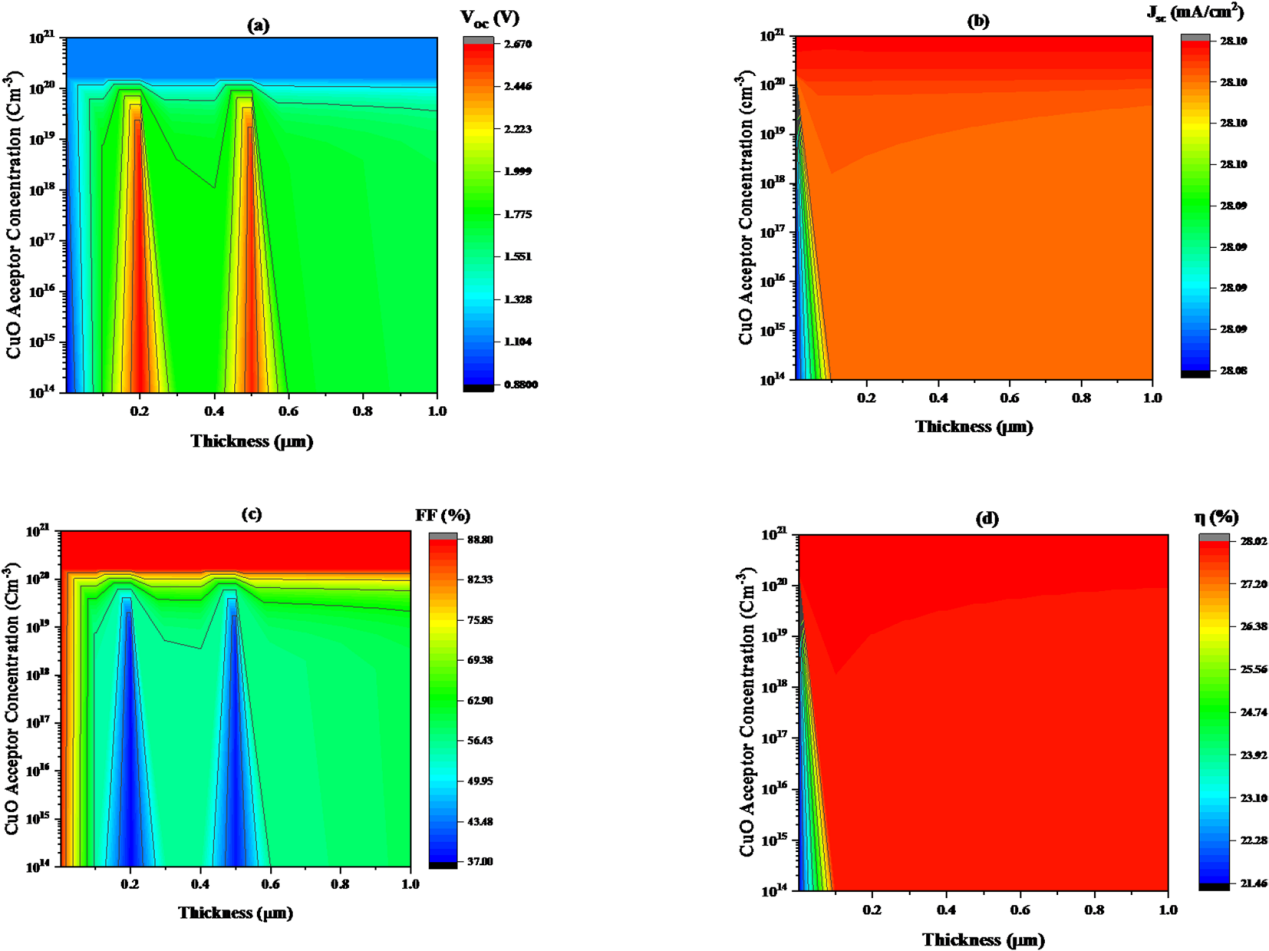
The variation of solar cell performance concerning carrier concentration and thickness of CuO HTL layer, keeping the band gap constant at 1.2 eV, where (**a**) V_oc_, (**b**) J_sc_, (**c**) FF, and (**d**) η.

**In** Fig. 4**(a)** The parameter analyzed is the V_oc_, which increases from approximately 2.223 V to 2.62 V as the CuO thickness increases from 0.1486 to 0.25237 μm and from 0.45516 to 0.4942 when the acceptor concentration rises from 1.039 × 10^14^ to 5.676 × 10^19^ cm^− 3^.

According to Fig. 4**(b)**, the plot illustrates how the short-circuit current density (J_sc_) changes with respect to both CuO acceptor concentration and thickness. For thin CuO layers (< 0.065 μm) and low donor concentrations (below 5.676 × 10^19^ cm⁻³), J_sc_ is relatively low (28.081 to 28.100 mA/cm²). The maximum J_sc_ value is roughly 28.114 mA/cm² at all thicknesses and at higher carrier concentration values (more than 1 × 10^20^ cm⁻³).

**In** Fig. 4**(c)** The parameter analyzed is the Fill Factor (FF), which increases from approximately 75.86–88.81% as the CuO thickness increases from 0.0027 to 0.046 μm when the acceptor concentration rises from 1.039 × 10^14^ to 1.021 × 10^21^ cm^− 3^.The FF was with the lowest value (37.6%) when thickness increases from 0.154 to 0.281 μm and from 0.461 to 0.521 μm when the acceptor concentration rises from 1.039 × 10^14^ to 5.676 × 10^19^ cm^− 3^.

As seen in Fig. 4**(d)**, the efficiency (η) typically increases when both thickness and acceptor concentration rise where it rich to the peak value (28.03%) when the thickness and acceptor concentration was 0.1 μm, 1 × 10^14^ cm⁻³, respectively. The η value remains as large as possible, no matter how much the thickness or acceptor concentration increases. The results confirm that the thickness factor and band gap are the most influential on the efficiency of the device.

#### J–V curve of cupric oxide (CuO) HTL layer

**Fig. 5:**
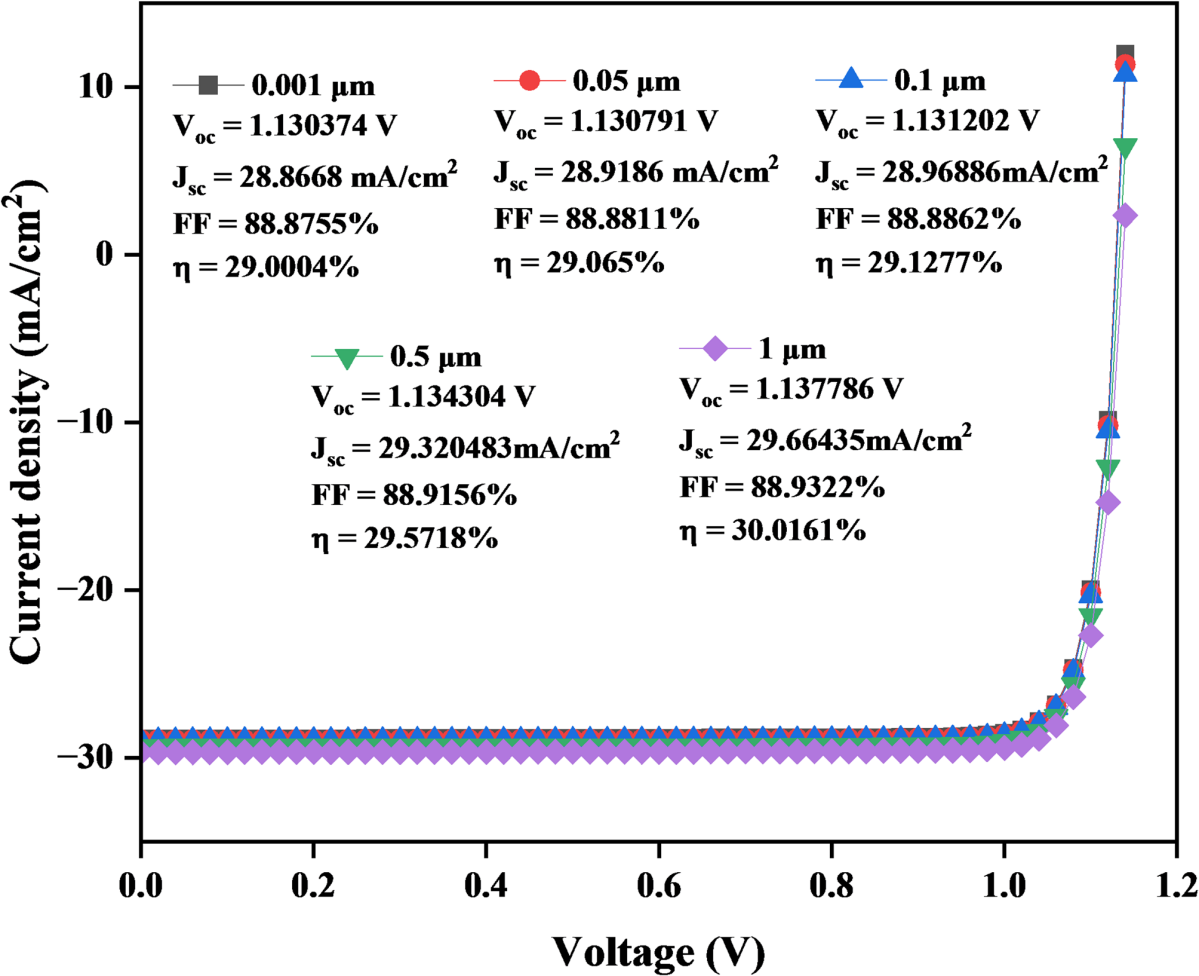
J–V Characteristics curve of a solar cell with varying of CuO layer thickness.

The performance of a solar cell with CuO acting as the hole transport layer (HTL) is depicted by the J-V curve. According to previous studies in this layer indicate that a band gap of that a band gap of 1.5 eV is the optimized results, and the thickness of the CuO ranges from 0.001 μm to 1 μm. Figure 5 indicates that the J_sc_ increases slightly from 28.8668 mA/cm² at 0.001 μm to 29.66439 mA/cm² at 1 μm, while the V_oc_ also increases slightly from 1.13037 V to 1.137786 V for thicknesses from 0.001 μm to 1 μm. The FF stays constant between 88.8755% and 88.9322%, contributing to the strong performance across different thicknesses. As the CuO thickness increases, the η improves slightly, from 29.0004% at 0.001 μm to 30.0161% at 1 μm, the J-V curves show a small increase in efficiency with the increased thickness.

#### External quantum efficiency (EQE) and the impact of cupric oxide (CuO) thickness, acceptor concentration, and band gap on it

Solar cells convert electromagnetic energy from sunlight into electrical energy by the photovoltaic effect. This conversion efficiency can be enhanced by improving photoelectric conversion efficiency, which is based on photo-generated current and diode properties. Quantum Efficiency is essential in the measurement of QE because photo augmentation effect and monochromatic radiation requirements are considered^41^. There are two types: External Quantum Efficiency (EQE), considering all incident photons, and Internal Quantum Efficiency (IQE), considering only absorbed photons (as shown in Eq. (1))^42^.

1 $$\:\varvec{E}\varvec{Q}\varvec{E}\:\left(\varvec{\%}\right)=\frac{\varvec{h}\varvec{c}}{\varvec{q}\varvec{\lambda\:}}$$

**Fig. 6:**
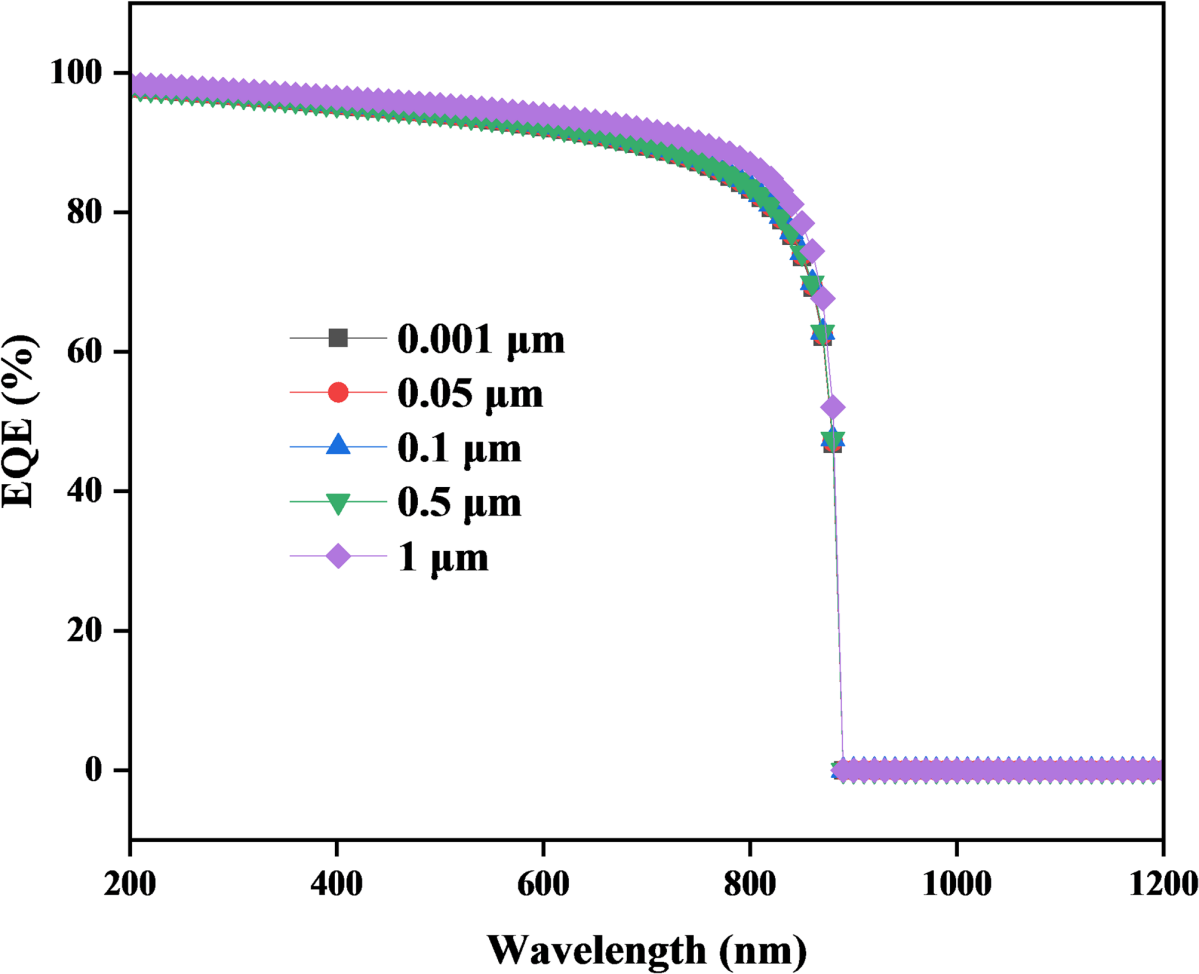
Shows that EQE is affected by cupric oxide thickness.

Figure 6 illustrates the relationship between External Quantum Efficiency (EQE) and wavelength for different CuO thicknesses (0.001 μm, 0.05 μm, 0.1 μm, 0.5 μm, and 1 μm). EQE rises sharply in the wavelength 200 nm with 100% EQE value, that indicating total photon-to-electron conversion^41^, However, for long wavelengths (more than 200 nm), shows a decrease of EQE reach to 0% on wavelength approximately 887 nm. That shows increasing the CuO thickness from 1 nm to 1000 nm leads to a slight increase in efficiency. However, we observed that this change has a minimal impact on the EQE, with only a slight improvement in performance.

### **Impact of copper zinc Tin sulfide as an absorber layer on solar cell performance**

Copper Zinc Tin Sulfide (CZTS) is one of the best materials for the absorber layer in solar cells, especially when simulated using SCAPS-1D, which is a one-dimensional tool. The ideal band gap of 1.3 eV of CZTS makes it possible to absorb a lot of visible light making this material be very useful in the photovoltaic applications. SCAPS-1D can change the composition of absorber layer thickness, doping concentrations as well as carrier dynamics to improve and enhance the efficiency and performance of CZTS solar cells. High absorption coefficient and absence of any toxicity of the CZTS components make such material environmentally friendly and effective for the next generations of solar technologies^43^.

#### The impact of the thickness of copper zinc Tin sulphide (CZTS) on V_oc_, J_sc_, FF, and η

The thickness of the CZTS absorber layer has a great influence on the performance of solar cells in terms of parameters such as V_oc_, J_sc_, FF, and η.

**Fig. 7:**
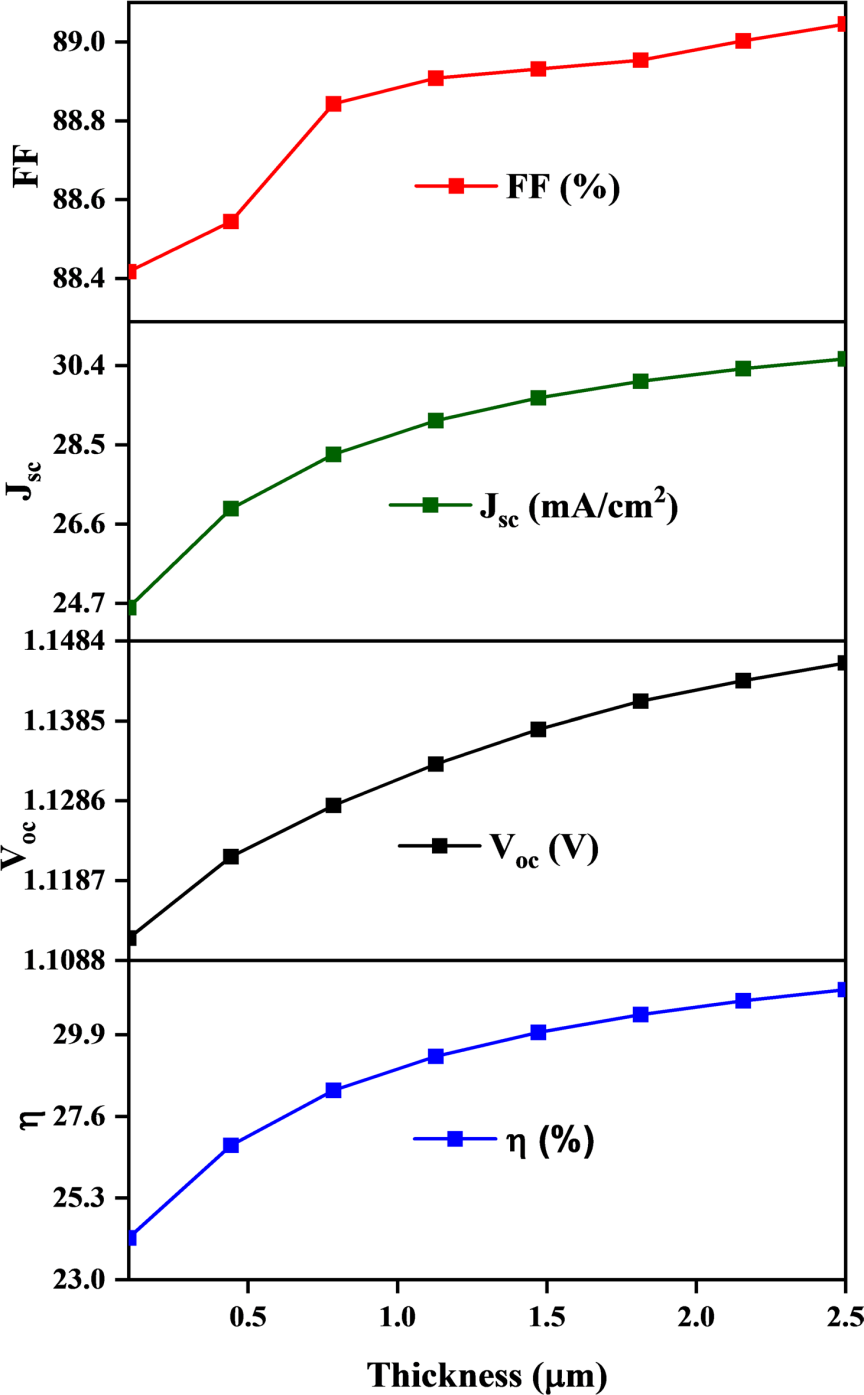
Shows the relationship between CZTS thin film thickness and the solar cell parameters.

**In** Fig. 7, while V_oc_ increases to reach (1.1461 V) with an increase in thickness due to reduced recombination losses, it decreases after a certain optimal value due to resistive losses. J_sc_ increases to reach (30.6601 mA/cm²) with the increase in thickness but decreases for thicker layers due to increased recombination. The optimal FF improves (89.05%) with increased thickness but is reduced if too thick due to resistive losses. Efficiency η increases and reaches up to (31.276%) with thickness up to an optimum thickness but is lower if it becomes too thick^43^. It is clear from the results that with increasing thickness, the efficiency increases, so we consider that the best thickness for the absorber layer is 2.5 μm.

#### Impact of copper zinc Tin sulfide (CZTS) thickness on band gap (E_g_) variation

This section examines how changes in the thickness and band gap of the CZTS affect the device’s performance. The band gap of CZTS varied between 1 and 2 eV, and the thickness ranged from 0.1 to 2.5 μm. Throughout this study, the acceptor concentration in the CZTS was held constant at 10^19^ cm⁻³, as detailed in Table 1.

**Fig. 8:**
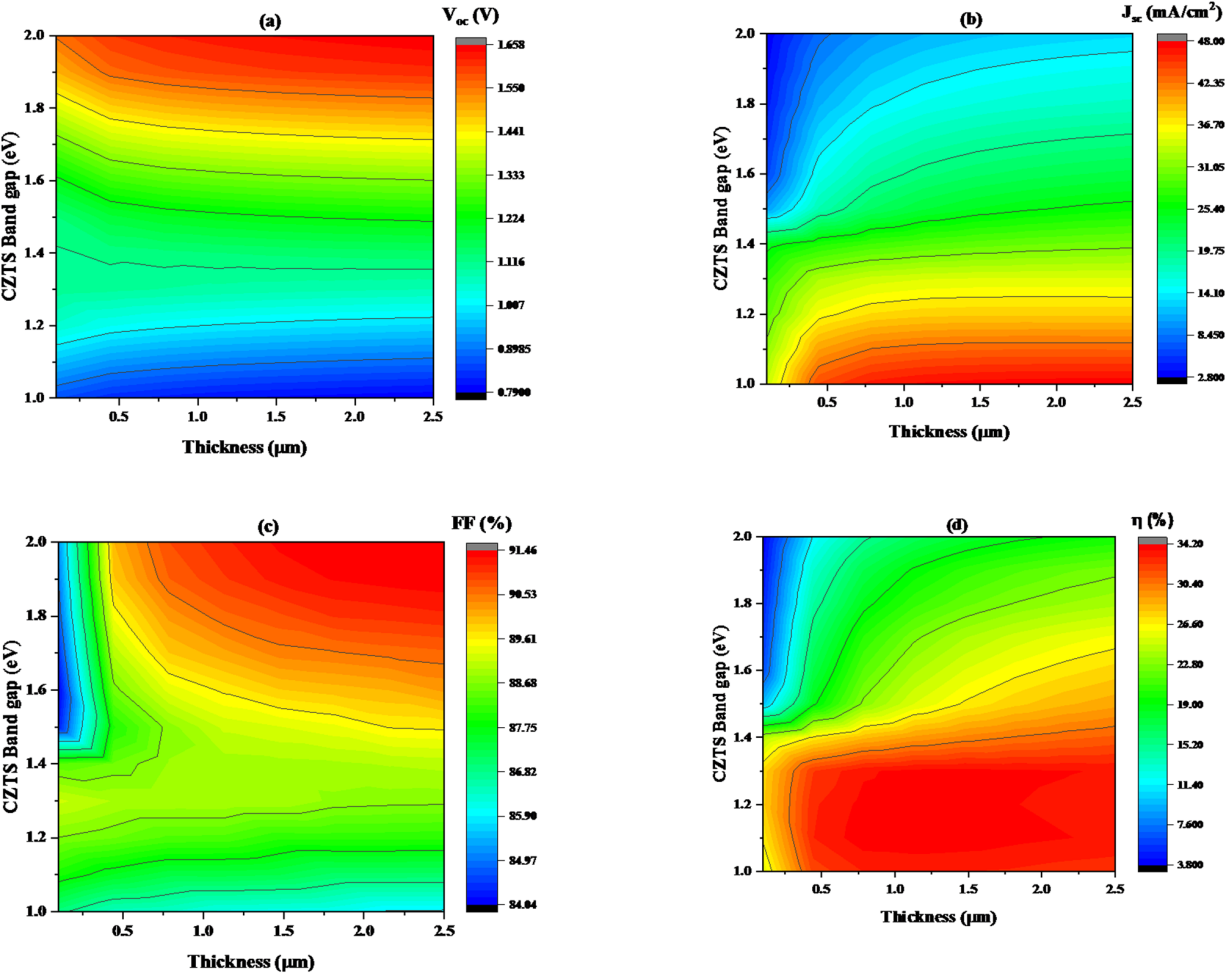
Illustrates the change in solar cell performance with variation in band gap and thickness of CZTS absorber layer for a given value of acceptor concentration 10^16^ cm^−3^, where (**a**) V_oc_, (**b**) J_sc_, (**c**) FF, and (**d**) η.

Figure 8**(a)** focuses on the open-circuit voltage (V_oc_), revealing an increase from around 1.45 V to 1.66 V as the band gap energy shifts from 1.71 to 2.0 eV. Interestingly, the V_oc_ remains largely unaffected by changes in CZTS thickness. This indicates that, across most of the tested range, the thickness has a negligible impact on the open-circuit voltage.

Figure 8**(b)** demonstrates that increasing the thickness of CZTS leads to an increase in short-circuit current density. J_sc_ initial values is 36.71 mA/cm² at a thickness of 0.2 μm; however, when the thickness increased, J_sc_ significantly increased. At thicknesses 2.5 μm, J_sc_ reached 48.01 mA/cm², which was the highest. However, the J_sc_ value decreased with increasing band gap.

As shown in Fig. 8**(c)**, that for CZTS films with thicknesses varying between 0.1 and 0.5 μm and band gaps in the range of 1.0 to 1.5 eV, the variation in performance parameters is insignificant. However, when the band gap and thickness of both increase, FF increases to a maximum value of 91.47%. This indicates that an optimal combination of increased thickness and band gap enhances the efficiency of charge collection and transport, contributing to improved overall solar cell performance.

Figure 8**(d)** demonstrates that increasing the thickness of CZTS leads to an increase in efficiency (η). η initial value is 26.61% at a thickness of 0.1 μm; however, when the thickness increased, η significantly increased. At thicknesses 2.5 μm, η reached 34.22%, which was the highest. On the other hand, the η value decreased with increasing band gap. The results show that the ideal thickness and band gap to achieve the highest efficiency in this device is 2.5 μm and 1.3 eV, respectively.

#### Impact of copper zinc Tin sulfide (CZTS) thickness on carrier (acceptor) concentration variation

The effect of changing the thickness and the concentration of CZTS absorber layer on the device’s performance has also been investigated in this part of the work. The carrier concentration of the CZTS was adjusted within the range of 10^14^ to 10^21^ cm^− 3^, and the thickness was varied from 0.1 to 2.5 μm. The band gap energy of CZTS was still constant at 1.5 eV, as shown in Table 1.

**Fig. 9:**
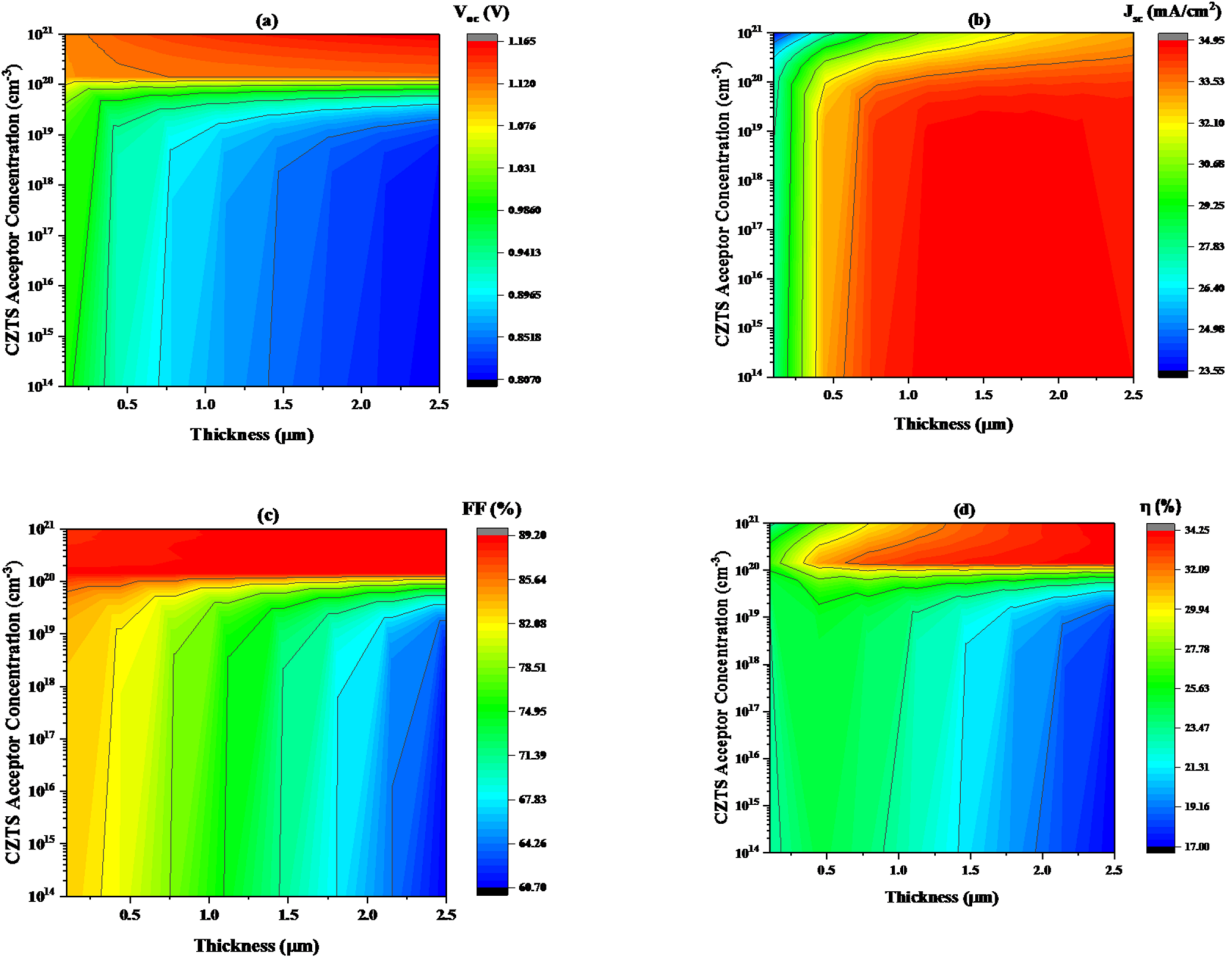
The variation of solar cell performance concerning carrier concentration and thickness of CZTS absorber layer, keeping the band gap constant at 1.3 eV, where (**a**) V_oc_, (**b**) J_sc_, (**c**) FF, and (**d**) η.

Figure 9**(a)** shows that the open-circuit voltage (V_oc_) decreases as the CZTS layer thickness increases when the carrier density is below 10^20^ cm^− 3^. However, when the carrier density exceeds 10^20^ cm^− 3^, V_oc_ values rise, reaching a maximum of 1.166 V as the thickness increases from 0.1 to 2.5 μm.

**In** Fig. 9**(b)**, the contour plot demonstrates how changes in thickness and carrier concentration impact the short-circuit current density (J_sc_) of CZTS films, where the maximum values of around 34.96 mA/cm^2^ occurring at a thickness of 0.35 to 2.5 μm and an acceptor concentration lower than 9.7 × 10^19^ cm^− 3^, highlighting that thicker films and acceptor concentrations lower than 9.7 × 10^19^ cm^− 3^ improve short-circuit current density performance in photovoltaic devices.

As shown in Fig. 9**(c)**. At acceptor concentrations below 8.9 × 10^19^ cm^− 3^, the FF values decreased with thickness improvement. In another hands the FF increased and reaches to peak value (89.21%) at acceptor concentrations more 8.9 × 10^19^ cm^− 3^ when thickness increased from 0.1 to 2.5 μm.

As seen in Fig. 9**(d)**, the efficiency (η) generally improves, a noticeable trend is observed whereas the thickness of the CZTS increases from 0.34 to 2.5 μm, especially beyond the point approximately 2.5 μm, and at acceptor concentrations more 1.09 × 10^20^ cm^− 3^ showing higher η values from (26.4–34.2%**)**. The findings indicate that the optimal thickness and acceptor concentrations for maximizing efficiency in this device are 2.5 μm and 1.0 × 10^20^.

cm^− 3^, respectively.

#### J–V curve of copper zinc Tin sulfide (CZTS) absorber layer

**Fig. 10:**
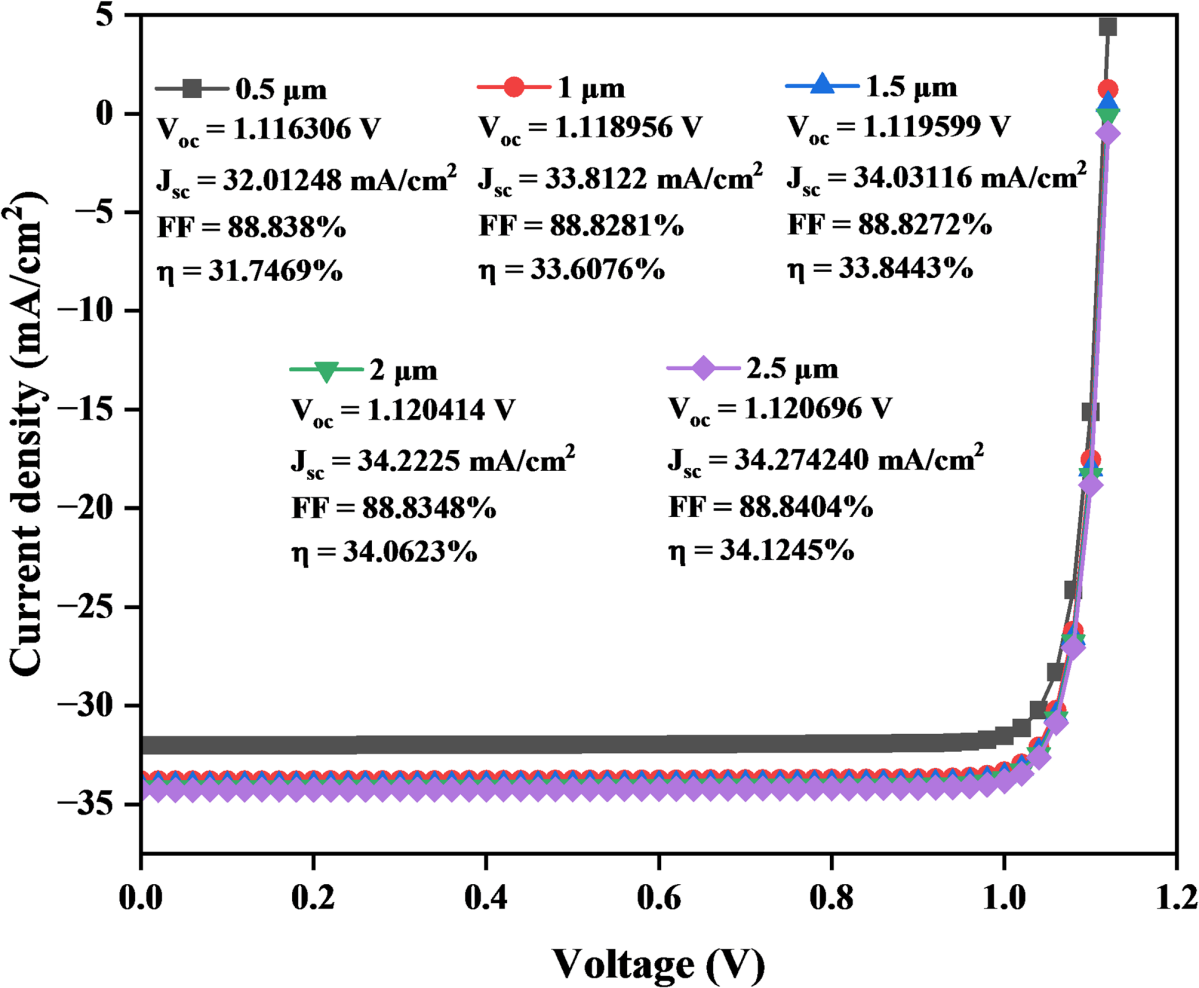
J–V Characteristics curve of a solar cell with varying of CZTS layer thickness.

Figure 10 indicates that the J_sc_ increases from 32.01248 mA/cm² at 0.5 μm to 34.274240 mA/cm² at 2.5 μm, while the V_oc_ also increases slightly from 1.116306 V to 1.1206 V for thicknesses from 0.5 μm to 2.5 μm. The FF decreases from 88.838% at 0.5 μm to 88.8272% at 1.5 μm and then increases slightly from 88.8348% at 2 μm to 88.8404% at 2.5 μm, contributing to the strong performance across different thicknesses. As the CZTS thickness increases, the η improves, from 31.7469% at 0.5 μm to 34.1245% at 2.5 μm.

#### EQE and the impact of copper zinc Tin sulfide (CZTS) thickness, acceptor concentration, and band gap on it

**Fig. 11:**
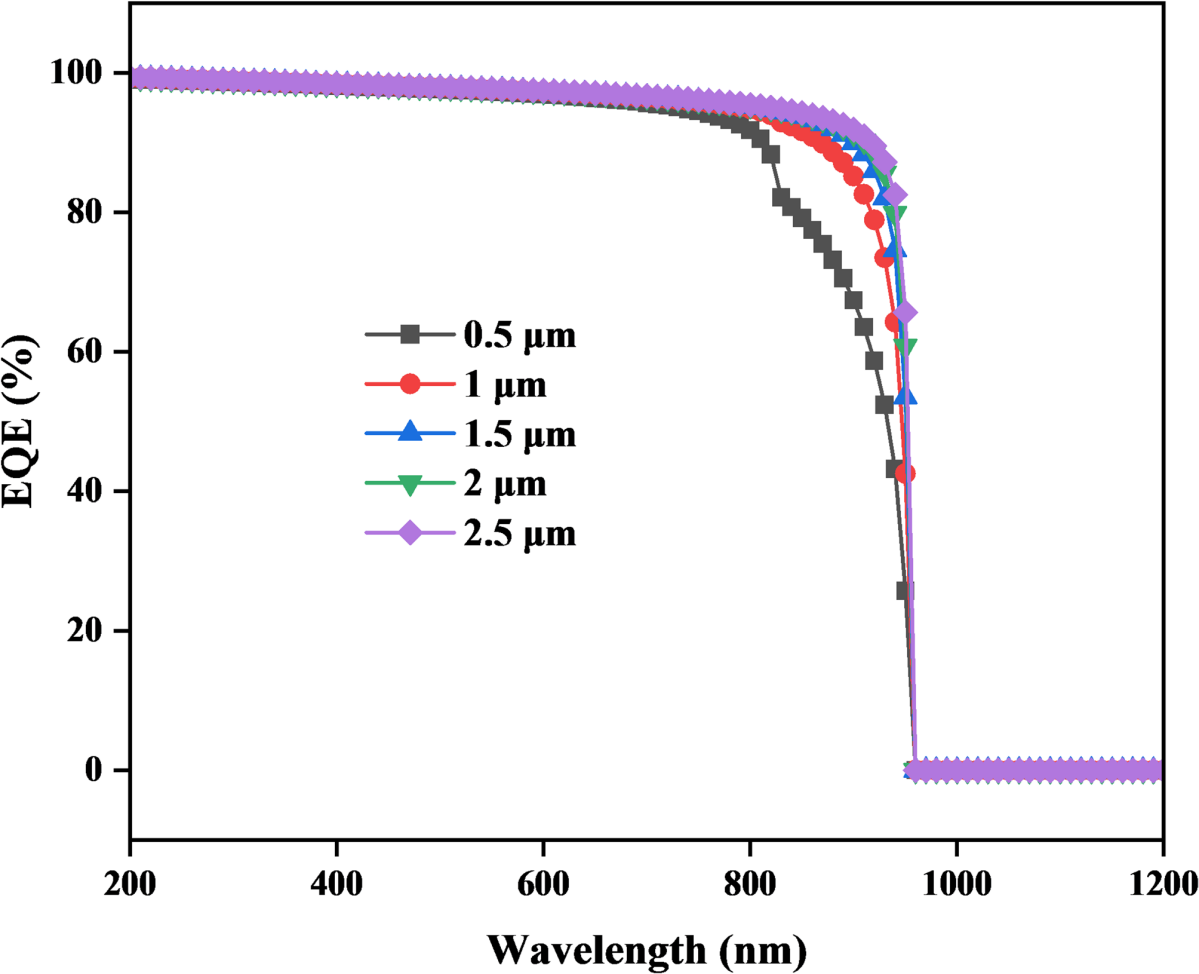
Shows that EQE is affected by copper zinc tin sulfide thickness.

Figure 11 illustrates the relationship between external quantum efficiency (EQE) and wavelength for different CZTS thicknesses (0.5 μm, 1 μm, 1.5 μm, 2 μm, and 2.5 μm). EQE rises sharply in the wavelength of 200 nm with a 100% EQE value, indicating total photon-to-electron conversion. However, for long wavelengths, approximately λ > 200 nm, it shows a decrease of EQE, where at lower thickness, EQE starts decreasing at small wavelengths, then at higher thicknesses, and then it reaches 0% at a wavelength of approximately 958 nm.

### Impact of ETL on the performance of the solar cell

Electron transport layers are needed to inject photogenerated electrons into n-type semiconductors, such as ZnO and SnO₂, which blocks the flow of holes that would create a short circuit to the FTO. The mostly used layer, however, is titanium dioxide (TiO₂), a very transparent material featuring strong carrier separation ability and environmental stability, for which ease of production makes it quite popular for use between the FTO conducting substrate and the absorber layer^44^.

#### Effect of titanium dioxide (n-TiO_2_) thickness on V_oc_, J_sc_, FF, and η

**Fig. 12:**
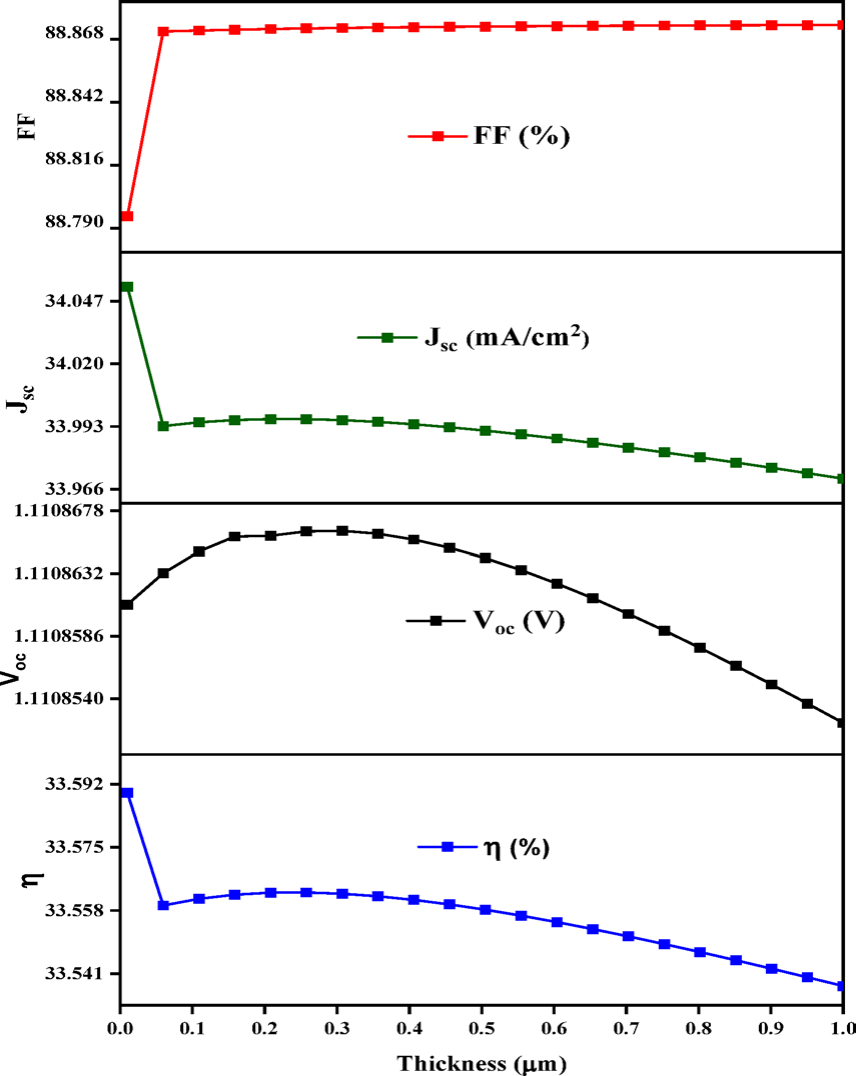
Shows the relationship between TiO_2_ thin film thickness and the solar cell parameters.

The TiO_2_ thickness is an essential challenge for developing an effective heterojunction solar cell since they have a considerable impact on the photo-produced excitons and carrier separation. The thickness in the case of ETL was studied from 0.01 mm to 0.1 nm, as illustrated in Fig. 12. The slight decrease in PCE from 33.56 to 33.53%, V_oc_ from 1.1108614 to 1.1108520 mA/cm^2^, J_sc_ From 33.9961 to 33.9705 mA/cm² and FF increase to reach 88.8720% and then remains constant. The results clearly demonstrate that as the thickness increases, the efficiency improves, leading us to conclude that the optimal thickness for the Electron Transport Layer is 0.001 μm.

#### Impact of titanium dioxide (TiO_2_) thickness on band gap (E_g_) variation

This section examines how changes in the thickness and band gap of the TiO_2_ affect the device’s performance. The band gap of TiO_2_ varied between 2.5 and 3.4 eV, and the thickness ranged from 0.01 to 0.1 μm. Throughout this study, the acceptor concentration in the TiO_2_ was held constant at 1 × 10^17^ cm⁻³, as detailed in Table 1.

**Fig. 13:**
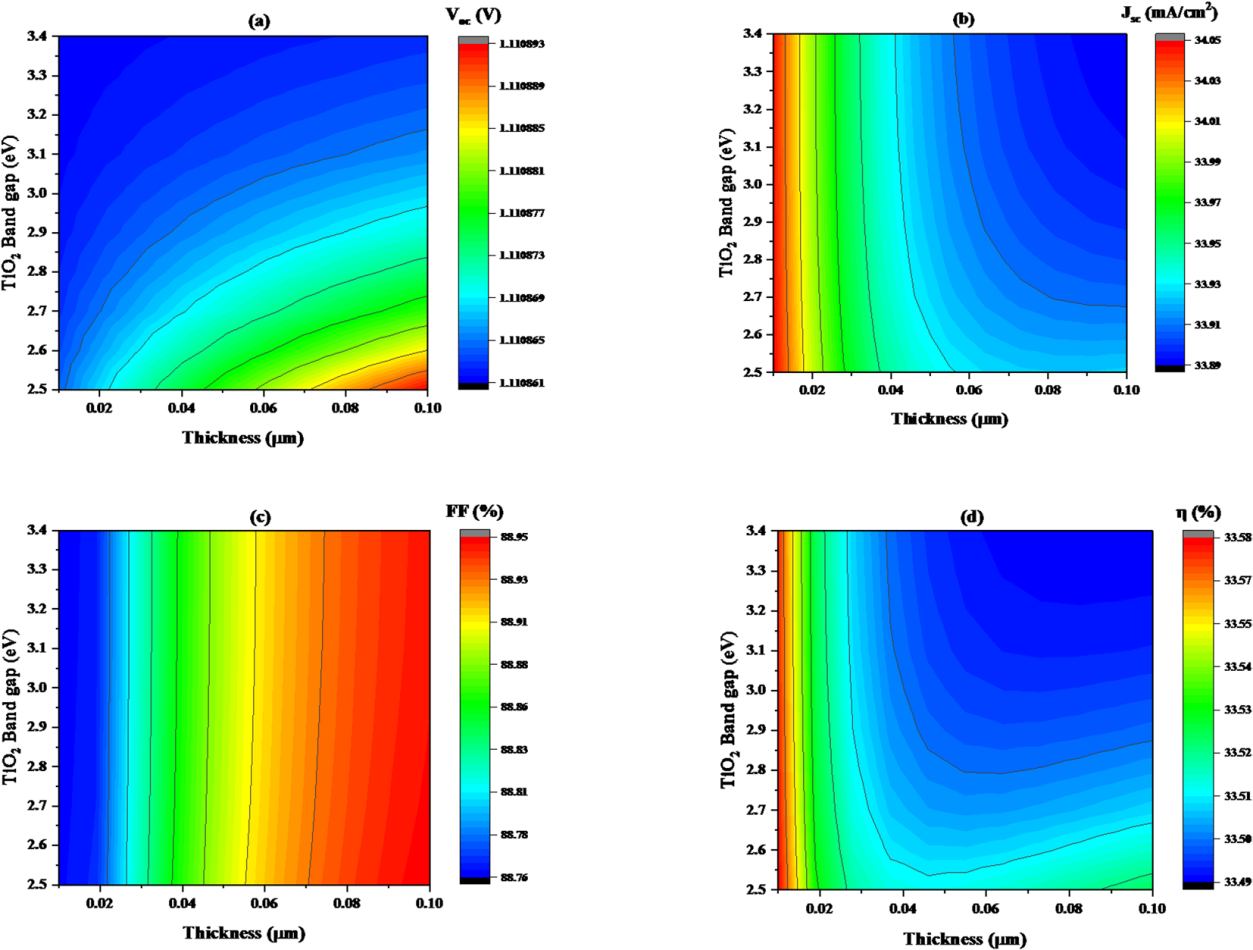
Illustrates the change in solar cell performance with variation in band gap and thickness of the TiO_2_ (as an ETL) for a given value of donor concentration 10^17^ cm^− 3^, where (**a**) V_oc_, (**b**) J_sc_, (**c**) FF, and (**d**) η.

Open-circuit voltage (V_oc_) is relatively constant for TiO_2_ films with band gaps ranging from 2.5 to 3.4 eV due to minimal impact of band gap changes on V_oc_, whereas thickness increases from 0.01 μm to 0.1 μm, the open-circuit voltage value rises insignificantly from 1.110884 V to 101,108,924 V As illustrated in Fig. 13**(a)**. At low thickness value, V_oc_ remains low, but as thickness increases, V_oc_ increases significantly, with values peaking at 1.1108885 V.

Figure 13**(b)** shows a relationship between thickness and band gap of TiO_2_ in the affection of short-circuit current density. J_sc_ increase in ranges from 34.041 to 34.052 mA/cm^2^, when thickness has a small value (less than 0.017 μm) with bad gap increasing. For larger TiO_2_ layers (more than 0.017 μm) and high band gap (over 2.5 eV), short-circuit current density drops to 33.895 mA/cm^2^.

Figure 13(**c)** expresses the change in fill Factor (FF) upon varying the band gap with thickness. As the thickness rises from 0.01to 0.1 μm, FF rises from around 88.90% to 88.951 when the thickness is around 0.0572 to 1.0 μm and the band gap is ranges from 2.5 to 3.4 eV. It is evident that the FF values increase as the thickness and band gap of the TiO_2_ absorber layer rise.

Figure 13**(d)** shows a relationship between thickness and band gap of TiO_2_ in the affection of efficiency (η). η increase in ranges from 33.551 to 33.582%, when thickness has a small value (less than 0.013 μm) with bad gap increasing. For larger TiO_2_ layers (more than 0.017 μm) and high band gap (over 2.5 eV), efficiency (η) drops to 33.49%. It is clear from the results that with increasing thickness, the efficiency increases, unlike the band gap, which is constant with increasing thickness. Therefore, we will consider that the best thickness is the one that has the greatest impact on the efficiency of the solar cell.

#### Impact of titanium dioxide (TiO_2_) thickness on carrier (donor) concentration variation

This section explores the effects of varying the N- TiO_2_ ETL layer’s thickness and carrier concentration to achieve an analysis of the effectiveness of the device. The N- TiO_2_ ETL’s carrier concentration was adjusted from 1E14 to 1E20 cm^− 3^, and its thickness was adjusted from 0.001 to 0.1 μm. The band gap energy of the TiO_2_ ETL was maintained at a constant value of 3.2 eV, as indicated in Table 1.

**Fig. 14:**
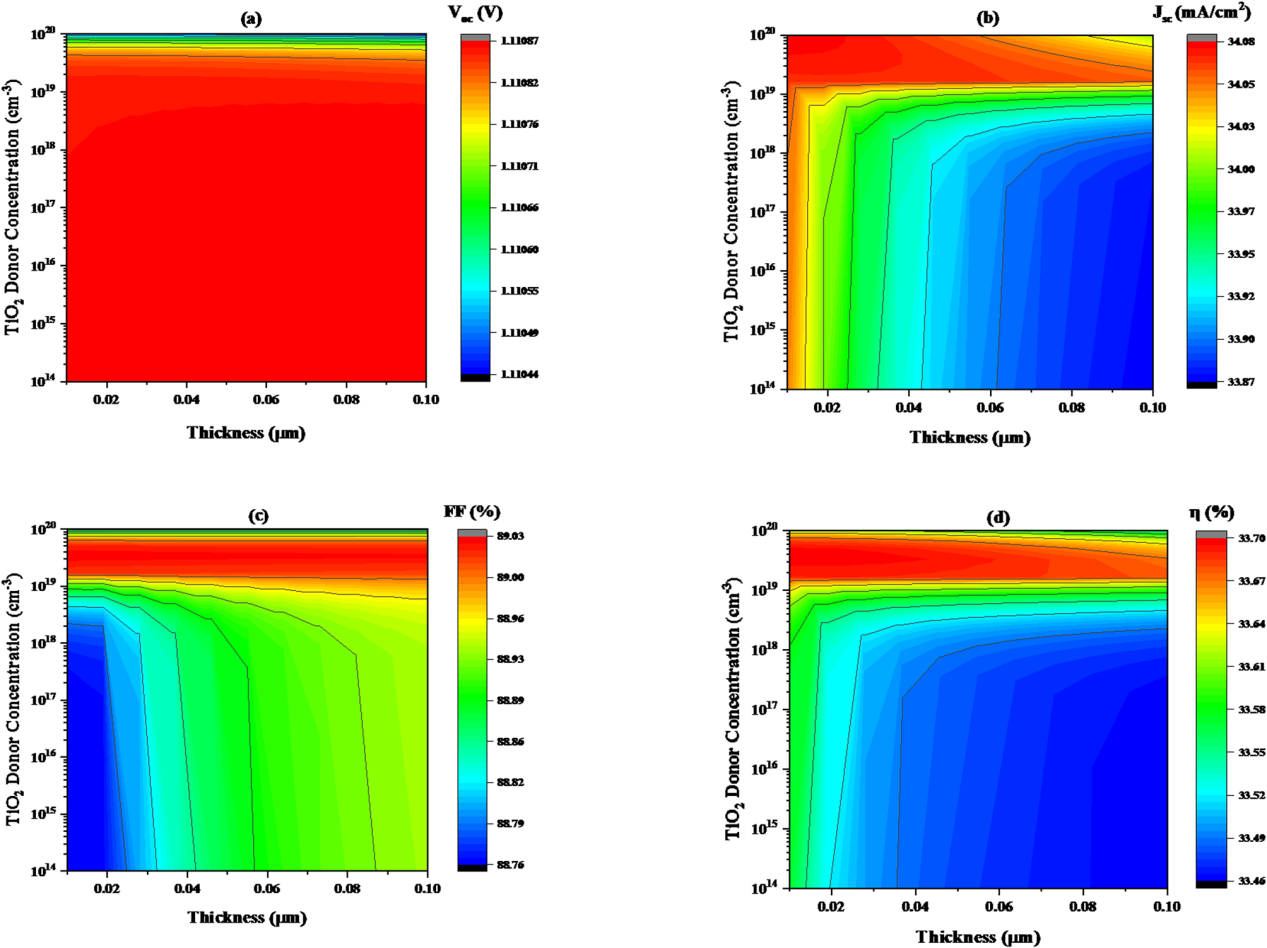
The variation of solar cell performance concerning carrier concentration and thickness of TiO_2_ (ETL layer), keeping the band gap constant at 3 eV, where (**a**) V_oc_, (**b**) J_sc_, (**c**) FF, and (**d**) η.

**In** Fig. 14**(a)** The parameter analysed is the open-circuit voltage (V_oc_), which increases from approximately 1.11076 V to 1.1087 V as the donor concentration more than 5.229 × 10^19^ cm^− 3^.Notably, the V_oc_ shows minimal variation when donor concentration below 5.229 × 10^19^ cm^− 3^ with increasing TiO_2_ thickness.

**In** Fig. 14**(b)** shows a relationship between thickness and donor concentration of TiO_2_ in the affection of short-circuit current density. J_sc_ increase in ranges from 34.0123 to 34.08 mA/cm^2^, when thickness has a small value (less than 0.012 μm) with donor concentration increasing. For larger TiO_2_ layers (more than 0.012 μm) and low donor concentration (below 1.2690 × 10^19^ cm^− 3^), short-circuit current density drops to 33.87 mA/cm^2^.

As shown in Fig. 14**(c)**. At acceptor concentrations more than 1.106 × 10^19^, the FF values increased reaches to peak value (89.03%) with thickness improvement. In another hands the FF decreased and drops to (88.76%) at donor concentrations below 1.106 × 10^19^ cm^− 3^ when thickness increased from 0.1 to 2.5 μm.

**In** Fig. 14**(d)** shows a relationship between thickness and donor concentration of TiO_2_ in the affection of efficiency (η). η increase in ranges from 33.65 to 33.70%, when thickness has a small value (less than 0.012 μm) with donor concentration from 1.2017 × 10^19^ cm^− 3^ to 8.1286 × 10^19^ cm^− 3^. For larger TiO_2_ layers (more than 0.012 μm) and low donor concentration (below 1.286 × 10^19^ cm^− 3^), efficiency drops to 33.46%. The results suggest that the ideal thickness and donner concentrations for achieving maximum efficiency in this device are 0.001 μm and above 1.0 × 10^19^ cm^− 3^, respectively.

#### J-V curve of titanium dioxide (ETL)

**Fig. 15:**
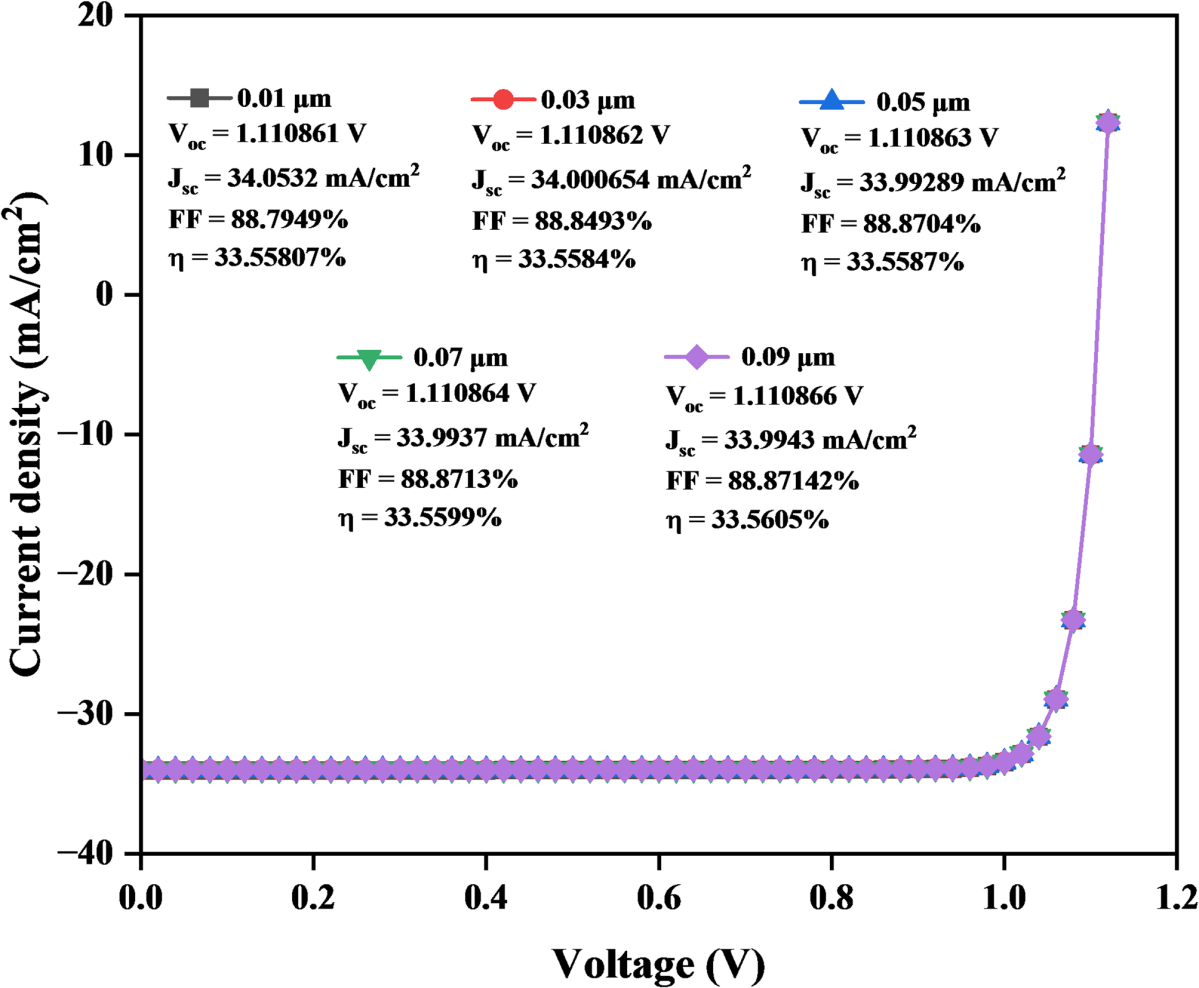
J–V Characteristics curve of a solar cell with varying of TiO_2_ layer thickness.

Figure 15 indicates that the J_sc_ decreases from 34.0532 mA/cm² at 0.01 μm to 33.9928 mA/cm² at 0.05 μm and then increases slightly from 33.9937 mA/cm² at 0.07 μm to 33.994 mA/cm² at 0.09 μm, while the V_oc_ stays constant between 1.110861 V and 1.110864 V for thicknesses from 0.01 μm to 0.09 μm. The FF is increasing slightly from 88.7949 to 88.8714%, contributing to the strong performance across different thicknesses. As the TiO_2_ thickness increases, the η improves slightly, from 33.55807% at 0.01 μm to 33.5606% at 0.09 μm.

#### External quantum efficiency and the impact of titanium dioxide (TiO_2_) thickness on it

**Fig. 16:**
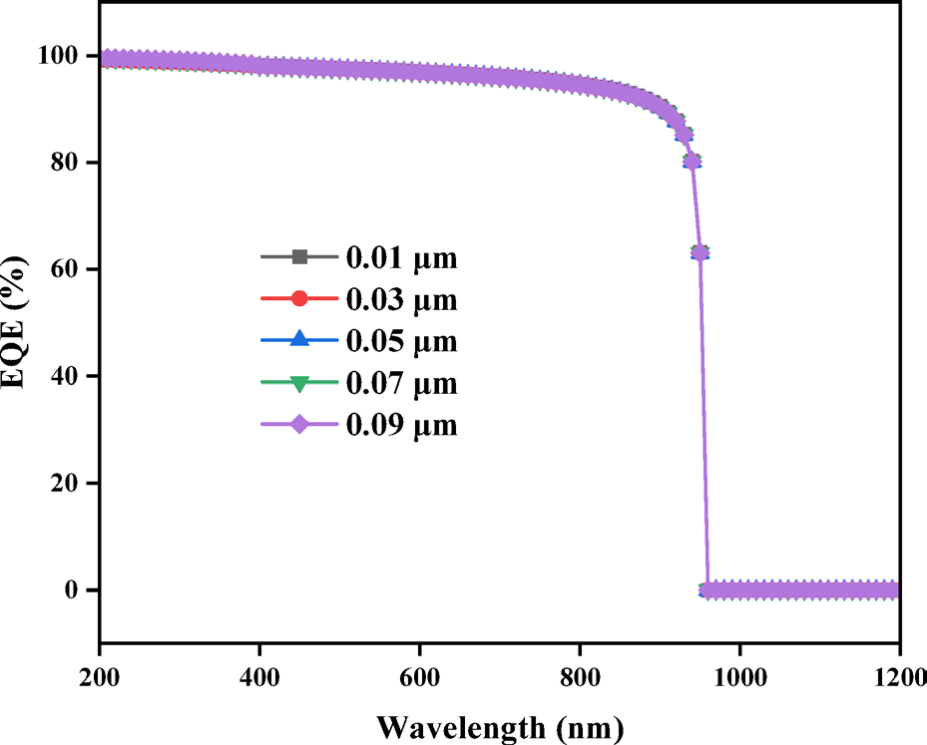
Shows that EQE is affected by Titanium dioxide thickness.

Figure 16 illustrates the relationship between External Quantum Efficiency (EQE) and wavelength for different TiO_2_ thicknesses (0.01 μm, 0.03 μm, 0.05 μm, 0.07 μm, and 0.09 μm). EQE rises sharply in the wavelength 200 nm with 100% EQE value, that indicating total photon-to-electron conversion^41^, However, for long wavelengths (more than 200 nm), shows a decrease of EQE reach to 0% on wavelength approximately 959 nm.

### Effect of the czts/tio₂ (absorber/ETL) and czts/cuo (absorber/HTL) interface layers defect

**Fig. 17:**
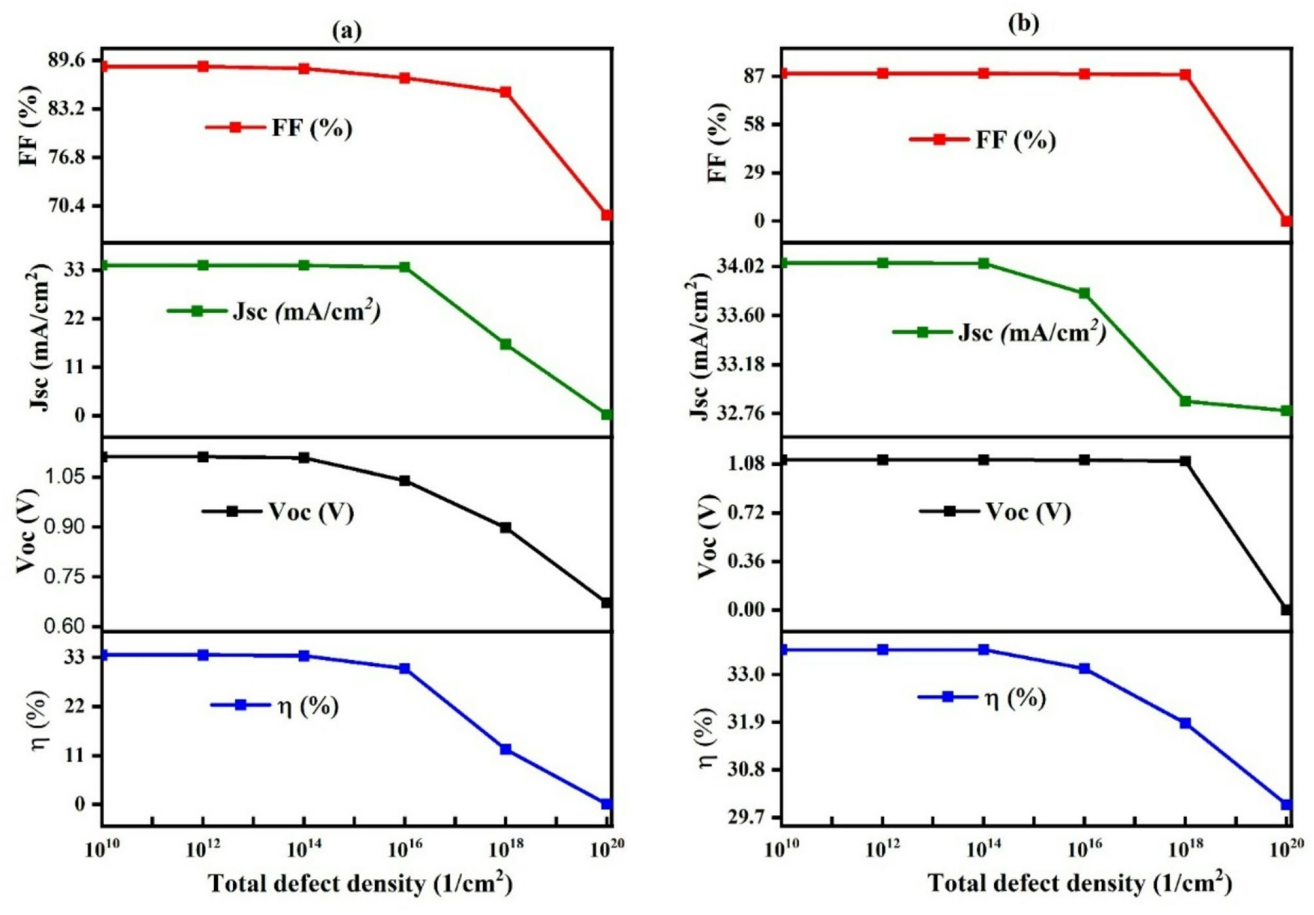
Impact of interface layer defect (**a**) CZTS/TiO₂(absorber/ETL) and (**b**) CZTS/CuO (absorber/HTL) Variation on Solar Cell Performance.

Defects at the CZTS/TiO₂ and CZTS/CuO interfaces significantly influence the performance of CZTS-based solar cells by impacting charge extraction and recombination. At the CZTS/CuO (absorber/HTL) interface, high defect densities introduce trap states and band misalignments that hinder hole transport and increase recombination, leading to reduced open-circuit voltage (V_oc_), fill factor (FF), and overall efficiency. Similarly, defects at the CZTS/TiO₂ (absorber/ETL) interface can cause unfavorable conduction band offsets and electron traps, which obstruct electron extraction, raise recombination rates, and diminish charge separation, further lowering V_oc_ and device output. Effective interface engineering and defect passivation are therefore essential to enhance photovoltaic performance. The total defect density at the CZTS/ CuO and CZTS/TiO₂ interface layers in the solar cell structure, ranging from 1 × 10¹⁰ to 1 × 10^20^ cm⁻², significantly affects the device’s performance, as shown in Fig. 17.

**In** Fig. 17**(a)**, increasing the defect density at the CZTS/TiO₂ (absorber/ETL) interface causes a pronounced decline in the overall performance of the solar cell. As the defect density increases from 1 × 10¹⁰ to 1 × 10²⁰ cm⁻³, the open-circuit voltage (V_oc_) drops significantly from 1.11 V to 0.671 V, the short-circuit current density (J_sc_) decreases sharply from 34.04 mA/cm² to just 0.216 mA/cm², the fill factor (FF) reduces from 88.79 to 69.18%, and the conversion efficiency (η) declines from 33.3 to 24.45%. This sharp deterioration is mainly due to the high density of interface defects, which introduce numerous trap states. These traps act as recombination centers for photo-generated charge carriers, leading to higher non-radiative recombination rates. As a result, fewer electrons are extracted, charge collection becomes inefficient, and the internal electric field weakens, which collectively reduces voltage, current, and overall efficiency^45^.

**In** Fig. 17**(b)**, the impact of interface defects at the CZTS/CuO (absorber/HTL) interface shows a more gradual behavior. The V_oc_ remains unaffected at 1.11 V up to a defect density of 1 × 10¹⁶ cm⁻³, after which it gradually declines and eventually falls to 0 V at 1 × 10²⁰ cm⁻³, indicating severe recombination loss. The J_sc_ stays nearly constant at 34.04 mA/cm² up to 1 × 10¹⁴ cm⁻³ and then slightly decreases to 32.78 mA/cm² at the highest defect density, suggesting that hole transport is less sensitive to initial defect increases but deteriorates at extreme levels. The FF remains steady at 87% up to 1 × 10¹⁸ cm⁻³ before dropping to 68.18%, and the efficiency holds around 33.85% until 1 × 10¹² cm⁻³ and then declines slowly to 29.99% at 1 × 10²⁰ cm⁻³. This trend indicates that the CZTS/CuO interface can initially tolerate a moderate defect density without major losses, but at high defect concentrations, recombination dominates, resulting in a substantial performance decline^39^.

### Comparative analysis of cuo, Cu_2_O, and cuscn as hole transport layers (HTLs) in CZTS-based solar cells

The performance of hole transport layers (HTLs) in CZTS-based solar cells significantly influences key parameters such as External Quantum Efficiency (EQE) and J-V characteristics. This section focuses on the comparative analysis of CuO, Cu_2_O, and CuSCN as HTLs, examining their impact on EQE and J-V curves. By evaluating how these materials affect charge transport, efficiency, and device performance, the most suitable HTL for enhancing the overall effectiveness of CZTS solar cells is identified. And a comparative analysis of different heterojunction CZTS-based solar cells including various HTLs Based Solar Cells, focusing on the impact of various device structures on V_oc_, J_sc_, FF, and efficiency (η). As shown in Table 3.

#### J–V curve analysis of cuo, cu₂o, and cuscn as HTLs in CZTS-based solar cells

**Fig. 18:**
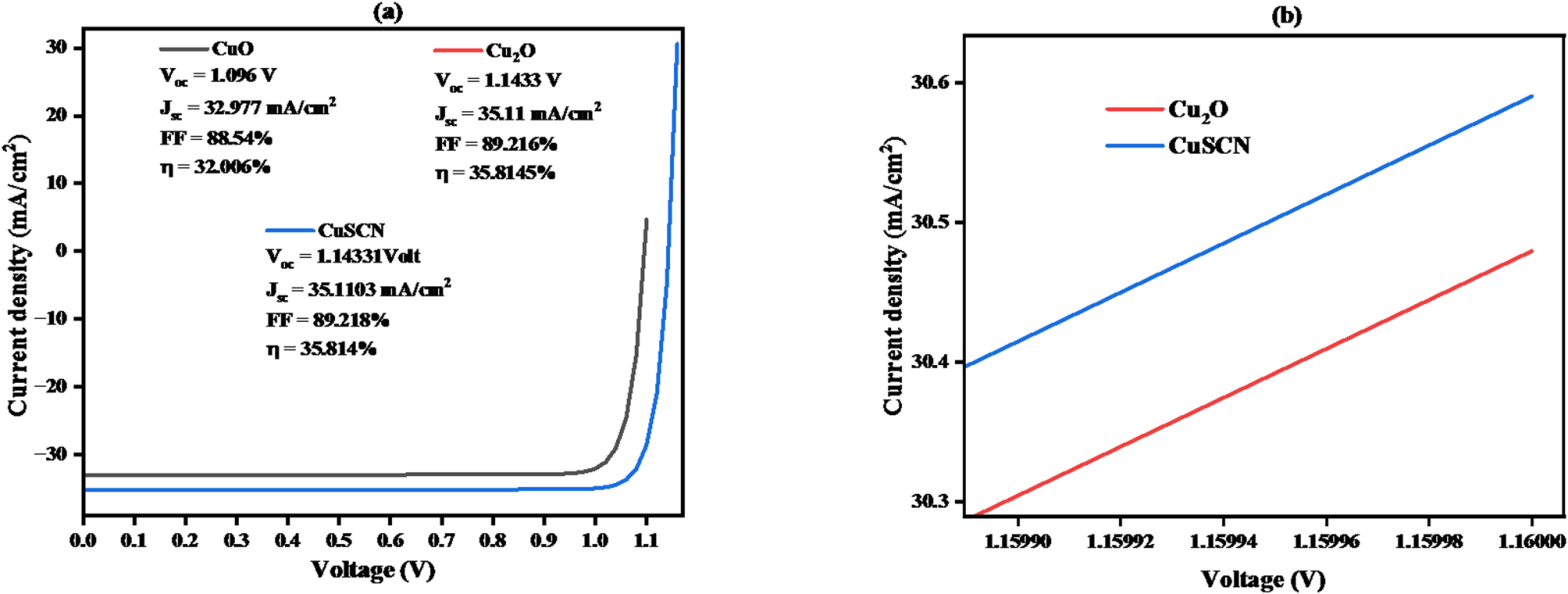
(**a**, **b**). J–V Curve of CuO, Cu₂O, and CuSCN as HTLs in CZTS-based solar cells.

The J-V curve in Fig. 18**(a**,** b)** compares the performance of CuO, Cu₂O, and CuSCN as hole transport layers (HTLs) in CZTS-based solar cells. Key parameters such as open-circuit voltage (V_oc_), short-circuit current density (Jsc), fill factor (FF), and efficiency (η) are presented for each material. CuO shows a V_oc_ of 1.096 V, J_sc_ of 32.977 mA/cm², FF of 88.54%, and an efficiency of 32.006%, making it the least efficient HTL. Cu₂O exhibits a higher V_oc_ of 1.1433 V, J_sc_ of 35.11 mA/cm², FF of 89.2166%, and efficiency of 35.8145%, making it the most efficient HTL. CuSCN performs well with a V_oc_ of 1.4331 V, J_sc_ of 35.1103 mA/cm², FF of 89.218%, and an efficiency of 35.814%. This analysis confirms that Cu₂O offers the best combination of hole mobility, energy alignment, and stability for CZTS solar cells, followed by CuSCN and CuO.

#### External quantum efficiency (EQE) analysis of cuo, cu₂o, and cuscn as HTLs in CZTS-based solar cells

**Fig. 19:**
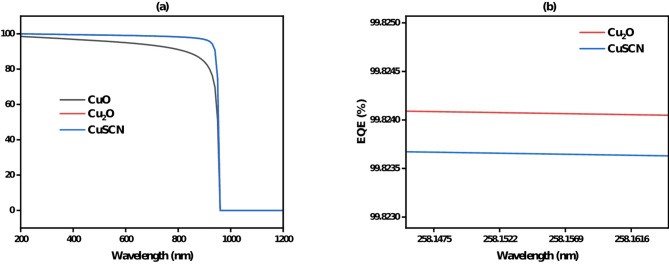
(**a**, **b**) External quantum efficiency (EQE) comparison of CuO, Cu₂O, and CuSCN as HTLs in CZTS-based solar cells.

Figure 19**(a)** shows the External Quantum Efficiency (EQE) for CuO, Cu₂O, and CuSCN over a range of wavelengths. Cu₂O performs the best, maintaining high EQE across the visible and near-infrared range, indicating excellent photon-to-electron conversion. CuSCN shows good performance but slightly lags behind Cu₂O, with moderate EQE values. CuO exhibits the lowest EQE, with a significant drop in efficiency as the wavelength increases. Figure 19(b) zooms in on the EQE values for Cu₂O and CuSCN around 258 nm, where Cu₂O shows a clear advantage with the highest efficiency, making it the best performing material compared to CuSCN.

**Table 3:** Compares the results of the current study with different heterojunction CZTS- including various HTLs based solar cells.

Device structure	Field of research	V_oc_ (V)	J_sc_ (mA/cm^2^)	FF (%)	η (%)	Reference
Mo/CZTS/CdS/ZnO	Theoretical	0.44	25.56	75.93	8.38	^46^
Glass/Mo/CZTS/SnS_2_ /ZnO	Theoretical	0.7178	26.9976	65.67	12.73	^47^
FTO/ZnS/CZTS/CuO/Au	Theoretical	0.9109	28.99	82.06	21.68	^48^
FTO/ZnO/CdS/CZTS/CZTSe/Mo	Theoretical	0.9355	28.2630	83.31	22.03	^49^
FTO/ZnS/CZTS/CuI/Au	Theoretical	1.029	29.16	81.17	24.36	^48^
FTO/ZnS/CZTS/CuSCN/Au	Theoretical	1.029	29.19	82.88	24.91	^48^
Pt/CZTSe/CZTS/WO_3_/ZnO	Theoretical	1.2	27.75	83.37	29.37	^50^
AI/ITO/C_60_/CZTS/SnS/Pt	Theoretical	1.24	27.03	89.96	30.18	^51^
FTO/ZnS/CZTS/Cu_2_O/Au	Theoretical	~ 1.19	~ 32.05	~ 83.37	~ 31.6	^48^
FTO/TiO_2_/CZTS/CuO/Au	Theoretical	1.1106	33.994	88.871	33.56	This Work

### Impact of operating temperature on J–V characteristics and key parameters of ideal TiO_2_/CZTS/CuO solar cells

**Fig. 20:**
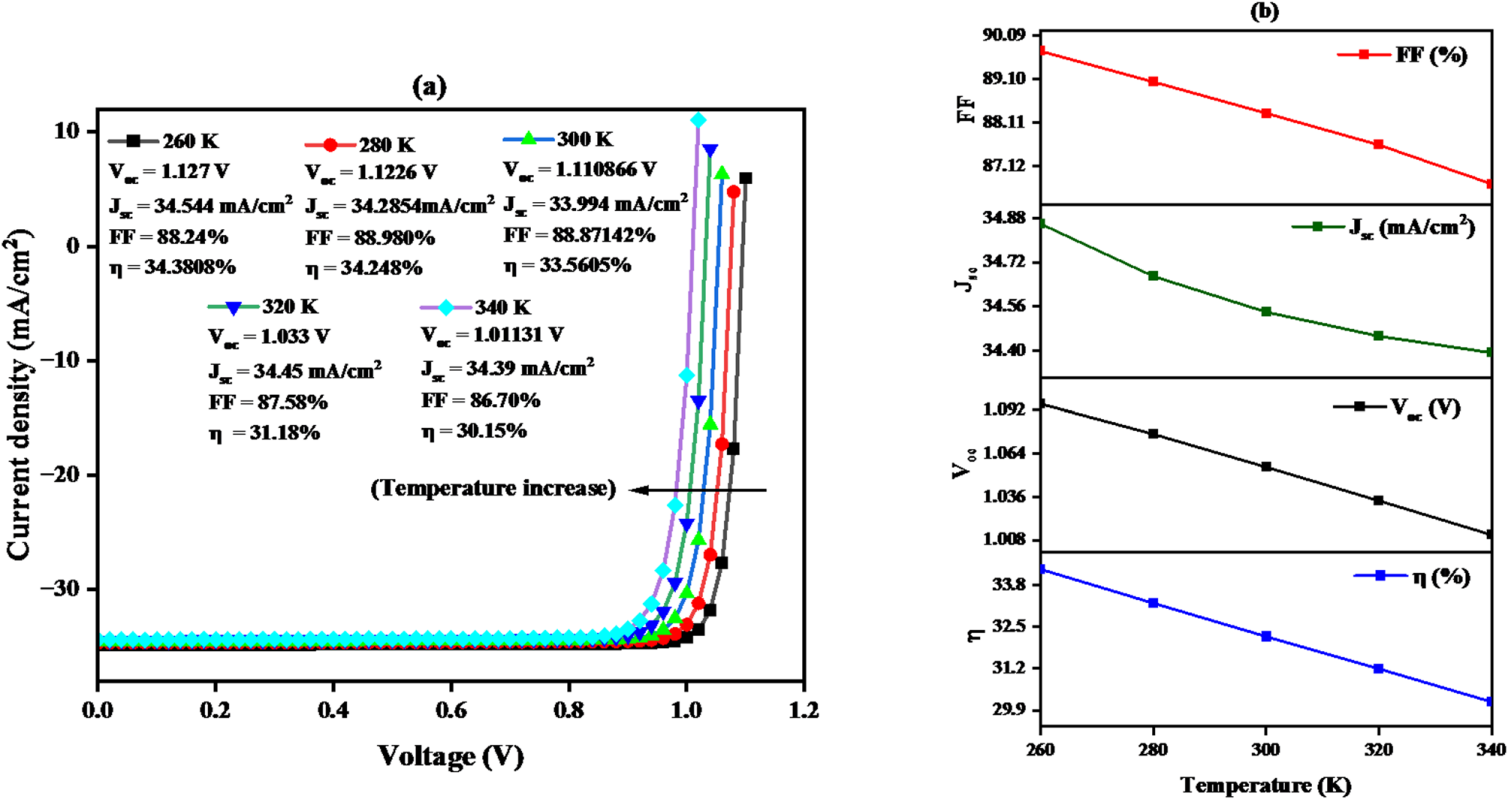
Shows the impact of operating temperature on the J–V curve (**a**) and the fundamental parameters of an ideal CuO/CZTS/ TiO₂ solar cell (**b**).

In this section, the influence of operational temperature on the JV curves and main parameters of the most efficient CuO/CZTS/ TiO₂ solar cell, depicted in Fig. 20**(a**,** b)**. It is easy to be remarked that an increase in the operational temperature produces a decline in V_oc_, FF and J_sc_ and implicitly in the overall efficiency (η). These values drop from 1.095 to 1.011 V, 89.72–86.70%, 34.85 to 34.39 mA/cm^2^, and 34.27–30.15% with an increase in temperature from 260 to 340 K, accordingly. This is because, with the rise in temperature, the chances of recombination among charge carriers before reaching the depletion region increase.

**Fig. 21:**
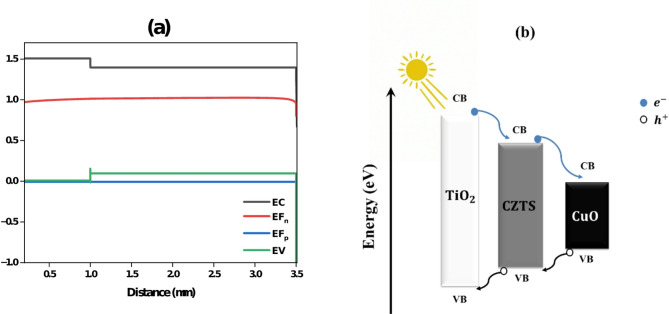
(**a**, **b**) Energy band diagram and charge carrier movement in a CZTS-based solar cell with CuO, CZTS, and TiO₂ layers.

Figure 21**(a)** illustrates the energy band structure of a multilayer semiconductor device, showing the conduction band (EC), valence band (EV), and the quasi-Fermi levels for electrons (EF_e_) and holes (EF_h_) across the device’s distance. At approximately 1 μm, a significant discontinuity in the conduction and valence bands indicates the interface between two materials with different bandgap energies. The electron quasi-Fermi level remains stable near 1 eV, suggesting efficient electron transport and minimal energy loss, while the hole quasi-Fermi level bends significantly, reflecting regions of hole accumulation and depletion that facilitate effective charge separation. These band offsets and Fermi level behavior help direct charge carriers, reduce recombination, and enhance the efficiency of optoelectronic devices such as solar cells^52^.

Figure 21**(b)** illustrates a CZTS-based solar cell structure where CuO serves as the hole transport layer (HTL), TiO₂ functions as the electron transport layer (ETL), and CZTS acts as the absorber layer. When sunlight is absorbed by CZTS (with a bandgap of 1.25 eV), it generates electron-hole pairs. The holes move towards CuO, which has a valence band aligned with the hole quasi-Fermi level in CZTS, allowing efficient hole transport. Meanwhile, the electrons are directed to the TiO₂ layer (with a bandgap of 3.2 eV), where the conduction band aligns with the electron quasi-Fermi level in CZTS, facilitating electron transport to the back contact. This structure ensures effective charge separation and transport, minimizing recombination losses and enhancing the overall efficiency of the solar cell^52,53^.

### Impact of different back contact electrodes on the performance of Kesterite-based solar cells

**Fig. 22:**
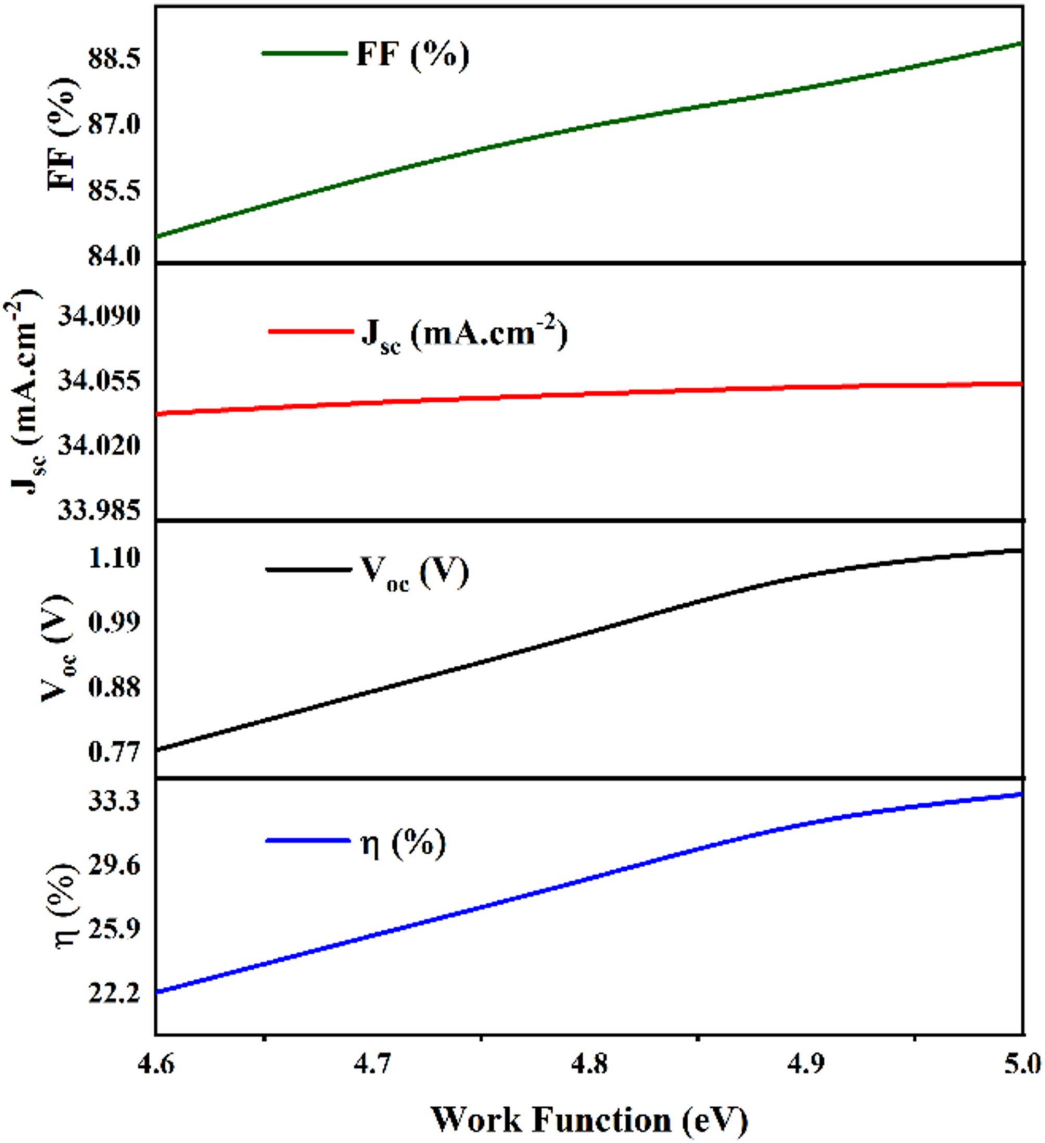
Shows the effect of work function on photovoltaic performance of Kesterite-based solar cell with different metal back contact electrodes.

The performance of kesterite-based solar cells depends significantly on the choice of the back contact electrode. Various metals, including graphene (4.60 eV), silver (4.74 eV), iron (4.81 eV), copper (5.00 eV), and gold (5.1 eV), were tested to determine which would optimize the device’s performance^54^. Figure 22; Table 4 clarify the work function of these metals and their associated photovoltaic parameters, which were simulated based on the initial device parameters provided in Tables 1 and 2. The results clearly show that gold (Au), with its work function of 5.1 eV, produced the most optimal performance for the kesterite-based solar cells. This was reflected in the device’s V_oc_ (Open Circuit Voltage) of 1.110866 V, J_sc_ (Short-Circuit Current Density) increases slightly to reach 34.054 mA/cm², FF (Fill Factor) of 88.87142%, and a PCE (Power Conversion Efficiency) of 33.5605%. These findings demonstrate that gold, due to its favourable work function and energy alignment with the kesterite-based solar cell layers, is the ideal choice for the back contact electrode in achieving enhanced solar cell performance.

**Table 4:** Photovoltaic performance of Kesterite-based solar cells with various metal back contact electrodes^54,55^.

Metal	Work function (eV)	η (%)	V_oc_ (V)	J_sc_ (mA/cm^2^)	FF (%)
Graphene	4.60E + 00	2.23E + 01	7.74E-01	3.40E + 01	8.45E + 01
Silver	4.70E + 00	2.55E + 01	8.74E-01	3.40E + 01	8.58E + 01
Iron	4.80E + 00	2.88E + 01	9.74E-01	3.40E + 01	8.70E + 01
Copper	5.00E + 00	3.37E + 01	1.11E + 00	3.41E + 01	8.89E + 01
Gold	5.10E + 00	3.38E + 01	1.12E + 00	3.41E + 01	8.89E + 01

## Conclusion and future work

Solar energy, harnessed from photovoltaic and concentrated solar, is a renewable and cleaner power source that propels sustainability in so many ways, ranging from applications, including homes, businesses, transportation, and space exploration. The efforts in the development of such elements relate to silicon, perovskites, thin films, and quantum dots. The goals of research provide optimisation of the bandgap, work out the feasibility for sustainability of materials, or scalability, stability, and ecological processes.

Copper zinc tin sulphide (CZTS) is found to be one of the most promising absorber materials for thin-film heterojunction solar cells thanks to its good properties when it comes to solar cells. It has a suitable band gap in the range of 1.4 to 1.5 eV, a quite large absorption coefficient (10⁴ cm⁻¹), and p-type conduction, which makes it suitable for use in solar cells. A solar cell model designed as “FTO/TiO_2_/CZTS/CuO/Au” was proposed, and numerical modelling and simulation were conducted using the SCAPS-1D software. Parameters from previous studies were incorporated to analyse the efficiency of kesterite-based solar cells.

The investigation focused on the photovoltaic properties of the proposed design, including open-circuit voltage (V_oc_), short-circuit current density (J_sc_), fill factor (FF), and overall efficiency (η). These properties were examined at 300 K under standard A.M. 1.5 G illumination conditions. The analysis also explored how variations in the thickness, band gap, carrier concentration, and operating temperature of the hole transport layer (HTL), electron transport layer (ETL), and absorber layer impact solar cell performance.

A key finding was the negative impact of increased operating temperatures on solar cell performance, with a noticeable decline in efficiency across all configurations as the temperature rose. After optimising all parameters, it showed the maximum efficiency to be 33.5605%. The values of open-circuit voltage, short-circuit current density, and fill factor were 1.110866 V, 33.994 mA/cm², and 88.87142%, respectively. These results prove the capability of the proposed model in developing high-efficiency solar cell technologies.

Future research in CZTS-based solar cells could focus on replacing CuO with Cu_2_O as the hole transport layer (HTL) to enhance hole mobility and improve bandgap alignment, thereby boosting efficiency and charge transport. CuO shows a V_oc_ of 1.096 V, J_sc_ of 32.977 mA/cm², FF of 88.54%, and an efficiency of 32.006%, making it the least efficient HTL. Cu_2_O, on the other hand, exhibits higher V_oc_ of 1.1433 V, J_sc_ of 35.11 mA/cm², FF of 89.2166%, and an efficiency of 35.8145%, marking it as the most efficient HTL. Furthermore, CuSCN shows good performance with V_oc_ of 1.4331 V, J_sc_ of 35.1103 mA/cm², FF of 89.218%, and efficiency of 35.814%. This analysis confirms that Cu_2_O offers the best combination of hole mobility, energy alignment, and stability for CZTS solar cells, followed by CuSCN and CuO. Additionally, exploring environmentally friendly materials, such as replacing gold (Au) with molybdenum (Mo) for back contacts, could reduce costs and environmental impact while maintaining performance, leading to more sustainable and affordable solar technologies.

## Data Availability

The datasets used and/or analyzed during the current study available from the corresponding author on reasonable request.
